# Impact of a massive earthquake on adherence to antiretroviral therapy, mental health, and treatment failure among people living with HIV in Nepal

**DOI:** 10.1371/journal.pone.0198071

**Published:** 2018-06-11

**Authors:** Bharat Singh Negi, Sunil Kumar Joshi, Minato Nakazawa, Tomohiro Kotaki, Anup Bastola, Masanori Kameoka

**Affiliations:** 1 Department of International Health, Kobe University Graduate School of Health Sciences, Hyogo, Japan; 2 Department of Community Medicine, Kathmandu Medical College, Kathmandu, Nepal; 3 Sukraraj Tropical and Infectious Disease Hospital, Kathmandu, Nepal; Hôpital Bichat-Claude Bernard, FRANCE

## Abstract

**Introduction:**

The April 2015 Nepal earthquake resulted in more than 8,700 deaths and 22,000 casualties including damage to health facilities. The impact of this situation on chronic conditions such as human immunodeficiency virus infection and acquired immune deficiency syndrome (HIV/AIDS) may become a long-lasting public health threat. Therefore, the objectives of this study were i) to assess the association of antiretroviral therapy (ART) adherence with mental health problems, and social behaviors, ii) to examine factors affecting treatment failure, and iii) to investigate changes in ART adherence and post-traumatic stress disorder (PTSD) among people living with HIV 6 and 12 months after the disaster.

**Methods:**

Study was conducted 6 months after the earthquake in 2015 with a sample size of 305 earthquake victims with HIV and followed after 12 months of the earthquake. A logistic regression analysis was used to examine relationships, while a paired *t*-test analysis was conducted to assess changes in adherence to ART and PTSD level at 6 months and 12 months after earthquake.

**Results:**

In the earthquake, 5.2% of the participants lost their family member. Approximately 44% of participants had earthquake-PTSD symptoms and 50% experienced HIV stigma. PTSD and HIV status disclosure were significantly associated with adherence to ART, while HIV stigma and religion were associated with treatment failure. PTSD and adherence levels to ART were significantly improved over the 6-month period.

**Conclusion:**

Awareness programs for general public to eliminate HIV stigma; promote psychosocial counseling to earthquake victims living with HIV in order to reduce PTSD will contribute to maintaining optimal ART adherence and to prevent treatment failure.

## Background

A pandemic of human immunodeficiency virus infection and acquired immune deficiency syndrome (HIV/AIDS) occurred over a short time period, and affected more than 36 million people worldwide by the end of 2015 [1], with Sub-Saharan Africa being the most affected region. Antiretroviral therapy (ART) represents lifesaving care that has improved survival rates and lowered the incidence of HIV-related complications among people living with HIV (PLHIV). ART has markedly reduced the number of deaths related to AIDS by more than 70% [2, 3] in developing countries.

Social and personal attributes such as HIV stigma, HIV seropositive status disclosure, depression, post-traumatic stress disorder (PTSD), social support, substance abuse, and age have been identified as important factors influencing ART adherence [4–8]. Stigmatized youths avoid social contact [9] and are less likely to disclose their HIV status, even to sexual partners [10], leading to unsafe sex [11, 12]. HIV status disclosure remains low in developing countries [13]. Complete HIV status disclosure has been associated with improved adherence to ART [14] and also contributes to early ART initiation. Late ART initiation leads to a higher risk of HIV drug resistance (HIVDR) [9]. ART adherence is a strong predictor of progression to AIDS and death [15–17], with more than 95% ART adherence being recommended.

Difficulties are often associated with accessing health services following disasters [18, 19]. After the great Hanshin earthquake, approximately 25% of victims encountered difficulties in receiving care or discontinued medical care [20]. A marked decline in HIV testing and new ART patient enrollment was observed in Haiti; however, the follow-up of registered ART patients remained very high after the earthquake [21]. Moreover, psychological issues have been reported under disaster conditions [22–25]. A previous study showed that, following an earthquake, the prevalence of PTSD was 58.2% after 2 months, 22.1% after 8 months, 19.8% after 14 months, 19.0% after 26 months, and 8.0% after 44 months [26]. PTSD and depression are common among victims after disasters [25, 27, 28].

HIV/AIDS has been a major public health threat in Nepal [29]. As of July 2014, there were 25,222 HIV cases in Nepal; more than 2,000 had already died [30]. Sex workers, MSM (man who have sex with men), injecting drug users, and migrant returnees were the major sources of HIV infection in Nepal, and HIV was the most prevalent among the 20–49 year age group (>85%) [30]. Although ART was introduced in Nepal in 2003 [31], only 11,000 PLHIV were on ART by 2016.

A massive 7.8 magnitude earthquake in Nepal on 25^th^ April 2015 resulted in more than 8,700 deaths and 22,000 casualties [32], leaving the infrastructure and health facilities severely damaged. The impact of this situation on chronic health conditions such as HIV/AIDS has the potential to become a long-lasting public health threat [33, 34]. Health service interruptions after a disaster are common in developing countries due to facility damage and the lack of resources and preparedness [35]. Following the earthquake in Nepal, people left their homes and gathered on the streets for more than one week due to the fear of aftershocks. The ART adherence status among PLHIV was not reported following the disaster. The irregularity of ART has been suggested to result in therapeutic failure [16, 17] and HIVDR [36].

Therefore, the objectives of the present study are: 1) to assess the association of ART adherence with PTSD, stigma, HIV status disclosure, and social support under post-disaster conditions, 2) to investigate factors affecting treatment failure, and 3) to examine changes in ART adherence and PTSD among PLHIV 6 and 12 months after the disaster.

## Methods

### Study population and setting

Study participants were PLHIV living in an earthquake-affected area in Nepal. Fourteen out of 75 districts were destroyed by the massive earthquake in Nepal. Among 12 ART service centers in earthquake-affected areas that provide ART services to approximately 3,000 PLHIV, 10 functional ART centers were selected. PLHIV visit ART centers periodically for their CD4 count, viral load examination, antiretroviral drugs, treatment of opportunistic infections, and counseling services.

Trained enumerators collected data in two stages. In the 1^st^ stage, 6 months after the earthquake, face-to-face interviews were conducted at ART centers between November and December 2015. Peripheral blood samples were simultaneously collected. In the 2^nd^ stage, a follow-up study was conducted 12 months after the earthquake in May and June 2016 through telephone interviews. The sample size of the present study was 305 PLHIV. This sample was calculated using the software Power and Precision (ver. 4) at a power of 80 and 95% confidence intervals considering the predictor variables PTSD (“Yes” response rate 28%) [37] and stigma (“Yes” response rate 75%) [38] as a reference with a 5% missing rate and 5% margin of error. Since the primary purpose of the present study was to assess the impact of the earthquake on PLHIV, we included PLHIV aged 18 years and older who, at the time of earthquake, had no ART treatment failure (no decrease in the CD4 count to the pre-therapy baseline, no HIV viral load >5000 copies/ml, or no serious opportunistic infections), were expected to be on ART for at least 6 months, and were on a first-line regimen. Their medical records were examined to confirm the above information. Most commonly used first-line ART regimens in Nepal were Zidovudine/Lamivudine/Nevirapine (ZDV/3TC/NVP) or Tenofovir/Lamivudin/Nevirapine (TDF/3TC/NVP).

PLHIV attending the ART centers were recruited on a non-random basis if they met the inclusion criteria. Random sampling was not feasible and time-consuming because few PLHIV who met the inclusion criteria visited the ART center each day. Ethical approval was obtained from the Ethical Committees of Kobe University Graduate School of Health Sciences (Ethical approval no. 434) and Nepal Health Research Council (Ethical approval reg.no. 274/2015). The objective of this study was clearly explained to every PLHIV asked for volunteer participation before written consent was taken for the interview and blood samples collected.

### Measurements

#### Adherence to ART

An adherence questionnaire was adopted from the Adult AIDS Clinical Trials Group (AACTG), and is widely used [39]. It consists of several self-reported questions, and in the present study, missed doses for the previous 4 days were enumerated. The missed dose percentage was calculated based on total dose in the previous 4 days. Adherence was categorized into two groups based on the non-missed dose percentage; suboptimal adherence, <95% and optimal adherence, ≥95%.

#### Treatment failure

Since sufficient information was not available to confirm treatment failure, we chose three criteria based on National Antiretroviral Therapy Guidelines 2012 of Nepal,: CD4 count <250, viral load >5000, and persistent serious opportunistic infections provided that they were regularly taking ART for at least one month before their laboratory blood test. We did not carry out viral load testing in this study; however, we collected these information from their clinical records at ART Center. If PLHIV met at least one of the above three criteria, we considered it to be treatment failure.

#### PTSD

The PTSD Check List–Civilian Version (PCL-C) was used to assess PTSD. This scale was already used in Nepal in previous studies [40, 41]. PCL-C is a standardized self-reported rating scale for PTSD consisting of 17 items that correspond to the key symptoms of PTSD. PCL-C is applied to any traumatic events such as political conflict-affected people or natural disaster victims. This scale contains self-administered questions on how much they have been affected by a symptom over the past week using a 5-point Likert scale ranging from 1 “not at all” to 5 “extremely”. All individual scores were added and categorized into 3 groups based on three tertiles; the first tertile indicated weak PTSD symptoms, the second tertile medium symptoms, and the third tertile strong symptoms. The internal consistency of the scale (Cronbach’s alpha) was 0.90 in the present study.

#### Perceived family support

Perceived family support includes different forms of emotional and instrumental services and assistance from family members within the previous year. Support was measured using the 10-item Nepali Family Support and Difficulty Scale [42] that was developed specifically for use in Nepal. Regarding each item, participants were asked to rate how true each statement was for their own family on a four-point Likert scale ranging from “Not at all” (0) to “All the time” (3). Individual scores were added and the total score was grouped into two based on a median value indicating support “No” and social support “Yes”. The internal consistency of the scale (Cronbach’s alpha) was 0.87 in the present study.

#### HIV stigma

The HIV stigma scale was based on the Jacoby scale [43] of perceived stigma, which contains 3 items, each of which uses a 0 (No) or 1 (Yes) response. The measurement of perceived stigma is subjective; it assesses the way PLHIV perceive themselves as being stigmatized. Individual scores of the items were added and the total score was divided based on a median value into two groups; stigma “No” and stigma “Yes”. Cronbach’s alpha for this stigma scale was 0.83 in the present study.

#### Socio-economic factors

Socio-demographic information was assessed using the Nepal Demographic Health Survey (NDHS) 2011 questionnaire [44]. Information on demographic characteristics such as age, sex, occupation, religion, education, income, and area of residence was collected. Disclosure issues, substance use, the loss of family members, and the loss of a house due to the earthquake were included.

Additionally, there was an undeclared Indo-Nepal border blockade imposed by the Indian government accusing Indian-origin Nepalese protesters living in the adjoining border area of being responsible for it. This blockade continued from September 26^th^, 2015 until February 4^th^, 2016, and resulted in an acute humanitarian crisis in Nepal. Since Nepal was dependent on India for the import of fuel and other items, including medicine, internal public transportation was strongly affected because a sudden fuel shortage occurred in the country. This effect of the border blockade on PLHIV was also recorded as a confounding factor. PLHIV were asked whether they were able to visit ART centers on time using public transportation.

### Statistical analysis

Descriptive statistics were performed for all variables using the chi-squared test. Fisher’s exact test was used for variables with a very low response frequency. Adherence to ART and treatment failure, the outcome variables, were evaluated as dichotomous variables. All other covariates were categorical. The relationship of adherence to ART and treatment failure with covariates was measured using a Multiple Logistic Regression Model with a *p*-value < 0.05 indicating the significance of differences. The overall fit of the model was assessed by the Hosmer and Lemeshow Goodness-of-Fit test. The 95% confidence interval and adjusted odds ratio were used to evaluate the relationships between covariates and outcome variables adjusting for other possible confounders. A multicollinearity test was performed in advance in order to avoid any interaction effects among covariates. The parametric paired *t*-test was adopted to measure differences in ART adherence and PTSD at two stages: 6 and 12 months after the earthquake. SPSS Software version 16.0 (SPSS Inc. Chicago) was used in all statistical analyses.

## Results

### General characteristics of study participants

Females were more likely than males to be widowed or divorced (39.4% vs 7.3%, *p* < 0.001) and less likely to have a higher education (29.9% vs 57.3%, *p* < 0.001). Approximately one fourth of participants were unemployed, and the unemployment rate among females was more than two-fold that among males (38.6% vs 17.4%, *p* < 0.001). Although overall substance abuse among participants was not common, the substance abuse rate was more likely to be very high among males than among females (1.6% vs 18.0%, *p* < 0.001) (Table 1).

**Table 1:** Characteristics of study participants (N = 305).

		Female	Male	Total	
		n (%)	n (%)	n (%)	*p*-value
**Age**					
	40 and younger	84 (66.1)	93 (52.2)	177 (58.0)	**0.021**
	>40	43 (33.9)	85 (47.8)	128 (42.0)	
**Married status**					
	Unmarried	4 (3.1)	31 (17.4)	35 (11.5)	**<0.001**^**a**^
	Divorced/Widowed	50 (39.4)	13 (7.3)	63 (20.7)	
	Married	73 (57.5)	134 (75.3)	207 (67.9)	
**Education**					
	Illiterate	40 (31.5)	21 (11.8)	61 (20.0)	**<0.001**
	Primary	49 (38.6)	55 (30.9)	104 (34.1)	
	Secondary & above	38 (29.9)	102 (57.3)	140 (45.9)	
**Religion**					
	Hindu	87 (68.5)	126 (70.8)	213 (69.8)	**0.013**
	Buddhist	22 (17.3)	43 (24.2)	65 (21.3)	
	Christian or others	18 (14.2)	9 (5.1)	27 (8.9)	
**Occupation**					
	Agriculture	30 (23.6)	35 (19.7)	65 (21.3)	**<0.001**
	Business	14 (11.0)	41 (23.0)	55 (18.0)	
	Company employee	14 (11.0)	44 (24.7)	58 (19.0)	
	Laborer	20 (15.7)	27 (15.2)	47 (15.4)	
	Others/Unemployed	49 (38.6)	31 (17.4)	80 (26.2)	
**Area of residence**					
	Rural	49 (38.6)	70 (39.3)	119 (39.0)	0.99
	Urban	78 (61.4)	108 (60.7)	186 (61.0)	
**Smoking**					
	No	109 (85.8)	84 (47.2)	193 (63.3)	**<0.001**
	Yes	18 (14.2)	94 (52.8)	112 (36.7)	
**Substance abuse**					
	No	125 (98.4)	146 (82.0)	271 (88.9)	**<0.001**^a^
	Yes	2 (1.6)	32 (18.0)	34 (11.1)	
**Loss of a house in the earthquake**					
	No	40 (31.5)	57 (32.0)	97 (31.8)	0.966
	Completely	66 (52.0)	90 (50.6)	156 (51.1)	
	Partial	21 (16.5)	31 (17.4)	52 (17.0)	
**Loss of a family member in the earthquake**					
	No	119 (93.7)	170 (95.5)	289 (94.8)	0.663
	Yes	8 (6.3)	8 (4.5)	16 (5.2)	
**Stigma**					
	No	60 (47.2)	93 (52.2)	153 (50.2)	0.456
	Yes	67 (52.8)	85 (47.8)	152 (49.8)	
**HIV disclosure**					
	Yes	125 (98.4)	169 (94.9)	294 (96.4)	0.195^a^
	No	2 (1.6)	9 (5.1)	11 (3.6)	
**PTSD**					
	No	55 (43.3)	116 (65.2)	171 (56.1)	**<0.001**
	Medium	43 (33.9)	50 (28.1)	93 (30.5)	
	High	29 (22.8)	12 (6.7)	41 (13.4)	
**Support**					
	Low	62 (48.8)	53 (29.8)	115 (37.7)	**<0.001**
	Medium	47 (37.0)	69 (38.8)	116 (38.0)	
	High	18 (14.2)	56 (31.5)	74 (24.3)	
**Treatment failure**					
	No	113 (89.0)	153 (86.0)	266 (87.2)	0.545
	Yes	14 (11.0)	25 (14.0)	39 (12.8)	
**Adherence to ART**					
	<95%	11(8.7)	13(7.3)	24(7.9)	0.827
	>95%	116(91.3)	165(92.7)	281(92.1)	
**Hospital admission in the last 6 months**					
	No	105 (82.7)	163 (91.6)	268 (87.9)	**0.03**
	Yes	22 (17.3)	15 (8.4)	37 (12.1)	
**Border blockade effect**					
	No	117 (92.1)	167 (93.8)	284 (93.1)	0.729
	Yes	10 (7.9)	11 (6.2)	21 (6.9)	

^a^Fisher’s Exact Test

### Consequences of the earthquake and social behaviors

Approximately 50% of participants lost their house completely during the earthquake. Moreover, 5.2% lost a family member at the same time. Similarly, approximately 44% had PTSD symptoms. Females were more likely than males to report strong PTSD symptoms (22.8% vs 6.7%, *p* < 0.001).

Social behavior towards PLHIV appeared to be stereotypical in that approximately 50% of participants reported being stigmatized by either family or non-family members, with females encountering more stigma than males (52.8% vs 47.8%, *p* > 0.05). Similarly, 48.8% of females reported that they got no or low social support, whereas this was 29.8% among males (*p* < 0.001).

### Factors influencing ART adherence and treatment failure

In a multivariate logistic regression analysis, the age group ≥40s was less likely to adhere to ART than the age group <40 (AOR: 0.3, 95% CI: 0.08–0.95; *p* < 0.042). Some religious minorities such as Christians were also less likely to adhere to ART than Hindus, a dominant group in Nepal. HIV disclosure correlated with adherence to ART. Participants who had not disclosed their HIV status to anyone were less likely to adhere to ART than those who had disclosed their HIV status. (AOR: 0.02, 95% CI: 0.01–0.19; *p* = 0.001). Moreover, participants who reported strong PTSD symptoms were less likely to adhere to ART than those who did not report strong PTSD symptoms (AOR: 0.2, 95% CI: 0.04–0.91; *p* < 0.001) (Table 2).

**Table 2:** Factors associated with suboptimal adherence (4-day missed dose count) (N = 305).

		AOR	95% CI	*p*-value
Variables			(Lower—Upper)	
**Sex**				
	Female			
	Male	0.9	(0.21–3.67)	0.868
**Age**				
	40 and younger			
	>40	0.3	(0.08–0.95)	**0.042**
**Married status**				
	Unmarried			
	Divorced/Widowed	0.7	(0.06–8.21)	0.791
	Married	0.5	(0.06–4.69)	0.571
**Religion**				
	Hindu			
	Buddhist	0.6	(0.15–2.66)	0.526
	Christian or others	0.2	(0.03–0.97)	**0.047**
**Area of residence**				
	Rural			
	Urban	0.1	(0.02–0.43)	**0.002**
**Lost a house in the earthquake**				
	No			
	Yes	1.7	(0.43–6.57)	0.451
	Partial	3.5	(0.50–24.63)	0.208
**Lost a family member in the earthquake**				
	No			
	Yes	1.7	(0.14–19.52)	0.688
**Disclosure**				
	Yes			
	No	0.1	(0.01–0.19)	**0.001**
**Support**				
	Low			
	Medium	0.3	(0.07–1.17)	0.08
	High	0.4	(0.06–2.36)	0.296
**PTSD**				
	No			
	Medium	2.9	(0.64–12.68)	0.167
	High	0.2	(0.04–0.91)	**0.038**
**Stigma**				
	No			
	Yes	0.6	(0.15–2.19)	0.411
**Border blockade effect**				
	No			
	Yes	0.2	(0.04–0.94)	**0.042**

AOR: Adjusted odds ratio; CI: Confidence Interval

Adjusted for family size, education, income, alcohol use, substance abuse, and hospital stay for a certain period of time

A multivariate logistic regression analysis revealed that treatment failure was associated with religion and HIV stigma. Religious minorities such as Christians were four-fold more likely to have treatment failure than Hindus (AOR: 4.0, 95% CI: 1.22–13.17; *p* = 0.022). Similarly, participants who perceived stigma were 3.2-fold more likely to develop treatment failure than those who did not (AOR: 3.2, 95% CI: 1.29–7.76; *p* < 0.012) (Table 3).

**Table 3:** Factors associated with treatment failure (N = 305).

		AOR	95% CI	*p*-value
Variables			(Lower—Upper)	
**Sex**				
	Female			
	Male	2.4	(0.82–6.69)	0.109
**Age**				
	40 and younger			
	>40	0.9	(0.38–2.00)	0.760
**Married status**				
	Unmarried			
	Divorced/Widowed	3.3	(0.63–16.64)	0.156
	Married	1.8	(0.45–7.07)	0.409
**Religion**				
	Hindu			
	Buddhist	0.9	(0.28–2.66)	0.799
	Christian or others	4.0	(1.22–13.17)	**0.022**
**Area of residence**				
	Rural			
	Urban	0.8	(0.36–1.97)	0.696
**Lost a house in the earthquake**				
	No			
	Yes	0.4	(0.16–1.20)	0.111
	Partial	1.1	(0.39–3.12)	0.834
**Lost a family member in the earthquake**				
	No			
	Yes	1.6	(0.31–8.66)	0.556
**Disclosure**				
	Yes			
	No	0.2	(0.01–2.23)	0.178
**Support**				
	Low			
	Medium	0.5	(0.20–1.45)	0.224
	High	1.4	(0.48–3.84)	0.557
**PTSD**				
	No			
	Medium	1.0	(0.41–2.38)	0.993
	High	0.9	(0.24–3.22)	0.851
**Stigma**				
	No			
	Yes	3.2	(1.29–7.76)	**0.012**
**Border blockade effect**				
	No			
	Yes	1.4	(0.36–5.56)	0.619

AOR: Adjusted odds ratio; CI: Confidence Interval

Adjusted for family size, education, income, alcohol use, substance abuse, and hospital stay for a certain period of time

### PTSD and adherence to ART 6 and 12 months after the earthquake

A follow-up study was conducted 6 months after the first data collection through a phone call. In the follow-up study, only 210 (69%) out of 305 participants were interviewed. A paired *t*-test was conducted to evaluate changes in PTSD and adherence between two points of time. The significant decrease in the mean of the PTSD score at 6 to 12 months after the earthquake was 8.23 (t: 9.19, SD = 12.88, 95% CI: 6.46–9.99; *p* < 0.001). Similarly, the significant increase in the mean of the adherence score at 6 to 12 months after the earthquake was 0.06 (t: -3.28, SD = 0.25, 95% CI: -0.09 –-0.02; *p* = 0.001). This result clearly indicates improvements in PTSD and an increase in adherence levels among PLHIV (Table 4).

**Table 4:** Changes in PTSD and Adherence to ART (N = 210).

	Paired Differences	Std. Deviation	Std. Error Mean	95% Confidence Interval		t	df	*p*-value
	Mean			Lower	Upper			
PTSD 6 –PTSD 12	8.23	12.88	0.90	6.46	9.99	9.19	206	**<0.001**
Adherence 6—Adherence 12	-0.06	0.25	0.02	-0.09	-0.02	-3.28	209	**0.001**

PTSD6: PTSD level after 6 months of earthquake; PTSD12: PTSD level after 12 months of earthquake; Adherence 6: adherence level after 6 months of earthquake; Adherence 12: adherence level after 12 months of earthquake

## Discussion

Our study on PLHIV in a post-disaster situation showed that suboptimal adherence to ART was associated with non-disclosure of the HIV status by PLHIV, strong PTSD symptoms, and an urban residence. Another result is that ART treatment failure under post-disaster conditions was associated with HIV-related perceived stigma and religion. Christians were four-fold more likely to have treatment failure than Hindus. Finally, significant differences were observed in PTSD and adherence to ART scores at two different time points (6 and 12 months after the earthquake), indicating significant improvements in PTSD and adherence levels.

To the best of our knowledge, adherence to ART and its relationship with HIV disclosure and PTSD under disaster conditions have not yet been examined. However, our result showing adherence to ART and its association with HIV disclosure is consistent with previous studies [14, 45] conducted in non-disaster areas. This result may be attributed to those who disclose their HIV status to their family or parents obtaining social support, which is a key factor for medical adherence. Another factor that correlated with ART adherence in the present study was PTSD. Approximately 13% of respondents exhibited strong PTSD symptoms and were less likely to adhere to ART than those who had no PTSD symptoms. However, in previous studies, HIV-related PTSD (unlike earthquake-PTSD) was not associated with adherence to ART [7, 46].

ART treatment failure in the 6 months following the earthquake was approximately 13% and correlated with HIV-related perceived stigma and religion. Regarding treatment failure, the odds of the perceived stigma group was more than three-fold higher than that of the non-stigmatized group; this result is consistent with a previous study conducted on immigrants living with HIV in Amsterdam [47]. Half of the participants in the present study reported perceived stigma. HIV stigma is a strong predictor of depression [48], which is, in turn, associated with ART adherence [49, 50]. Therefore, HIV stigma has an indirect association with treatment failure. Our results also showed that Christians were more likely to report treatment failure than Hindus. The reason for this may be that Christianization is increasing in Nepal and mainly targets people with severe health conditions by telling them that praying to God is the only option that will help them [51]. Therefore, many PLHIV converted to Christianity and started praying instead of adhering to ART. Another reason may be discrimination towards Christians due to their different religious ideology.

Drug resistance analysis, which is discussed in separate paper [52], shows that in the 6 months following the earthquake, 22% of treatment-failure participants (7/32) had at least one major drug resistance mutations (DRM). Comparatively, low prevalence of major DRMs to reverse transcriptase inhibitors was previously reported in Nepal [53] under non-disaster conditions. This result suggests that the impact of the earthquake also played a role in increasing the drug resistance percentage.

Our study also showed changes in adherence to ART and PTSD levels at two-time points: 6 and 12 months after the earthquake. Significant progress in adherence to ART and PTSD was reported. The mean PTSD score decreased from 35.83 to 27.43. Previous studies based on the general population were also consistent with our results showing that PTSD scores decreased after a certain period of time following the earthquake [26, 54].

This study has some limitations. The adherence level to ART was based on missed doses in the previous 4 days; adherence to ART immediately after the earthquake was not assessed. Approximately 13% of participants had treatment failure, with 22% of these having ART DRMs [52], indicating that they did not maintain optimal adherence after earthquake for a period of time. Conditions soon after the earthquake were terrible, with frequent aftershocks occurring. People were afraid and remained outside in temporary houses for weeks. Under these conditions, PLHIV faced challenges to continue ART. Another limitation is that most of the information obtained in the present study was cross-sectional in nature; therefore, we cannot confirm a cause and effect relationship. Furthermore, the lost to the follow-up rate was high. A follow-up was performed through phone calls; however, many participants had changed phone numbers, networking issues occurred in the area, which was a remote mountainous region, two PLHIV died, and some declined the interview.

Despite these limitations, this study has several strengths. To the best of our knowledge, this is the first study of its kind to focus on adherence to ART, PTSD, and the treatment failure due to an earthquake among PLHIVs residing in a disaster and resource-limited area. Additionally, our results are important for policy implications in resource-limited developing countries during or after disasters. The present results also disclosed changes in ART adherence and PTSD levels over time among earthquake victims living with HIV/AIDS.

In conclusion, the present study showed that PTSD and disclosure of the HIV status correlated with adherence to ART, while HIV stigma and religion were associated with treatment failure among PLHIV residing in an earthquake-affected area in Nepal. PTSD and adherence levels to ART positively progressed over the 6-month time period; however, the appearance of a very high rate of DRMs posed a threat to PLHIV residing in the earthquake-affected area [52]. Concerned authorities need to promote awareness activities to eliminate social stigma and enhance psychosocial counseling, thereby reducing PTSD and mental health issues that may ultimately maintain optimal adherence levels to ART and reduce ART drug resistance issues in earthquake-affected areas.

## Supporting information

S1 File HIV GUIDELINE of Nepal 2012.pdf. (PDF)
