# Supplementary material for: Impact of a massive earthquake on adherence to antiretroviral therapy, mental health, and treatment failure among people living with HIV in Nepal
Source: PLoS One. 2018 Jun 11;13(6):e0198071. doi: 10.1371/journal.pone.0198071 (PMC5995447; doi:10.1371/journal.pone.0198071)
Supplement: S1 File — (PDF) [file pone.0198071.s001.pdf]

National Antiretroviral Therapy Guidelines

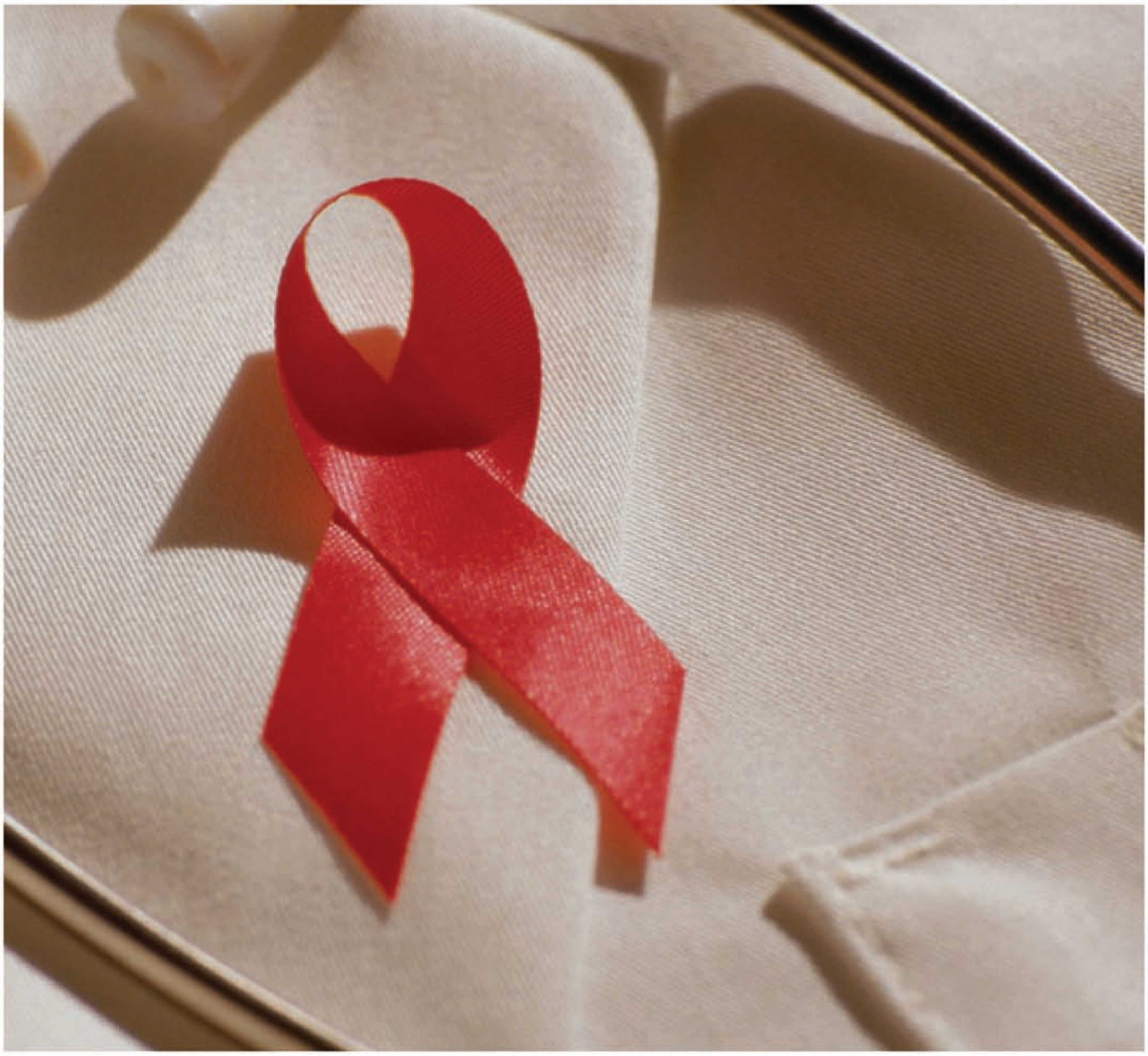

Government of Nepal
Ministry of Health and Population
National Centre for AIDS and STD Control (NCASC)
Teku, Kathmandu, Nepal
May, 2012

Copyright @ National ART Guidelines

Medical knowledge is constantly and rapidly changing, particularly in relation to HIV /AIDS. Readers are strongly advised to confirm that the information (especially with regards to drug doses and usage) complies with the latest standards of practice.

Foreword

HIV/AIDS remains a very important social and public health problem in the South Asian region. Over the years the National Center for AIDS and STD Control has done much to curb the spread of the virus through the introduction of prevention, treatment, care and support programs. Introduction of highly active antiretroviral therapy to eligible PLHIV since 2004 and establishment of prevention of mother-to-child transmission of HIV infection programs are some of these initiatives which have contributed a lot in saving lives of PLHIV. The national response to HIV/AIDS is guided by "HIV and STI Policy" 2010 and "National HIV/AIDS Strategy 2011-2016," which uses the principles of universal access.

Nepal with adult (15-49 years) HIV prevalence of 0.33% and approximately 55,626 people estimated living with HIV, our HIV caseload is hardly modest. While preventive efforts to bring down prevalence levels are ongoing, the importance of treatment, care and support of those that have already been infected cannot be over emphasized. The government of Nepal has made an effort to rapidly scale up antiretroviral therapy, so that treatment may be readily accessible to those in needs. Antiretroviral therapy is available today at 35 ART centers in the country and we intend to rapidly scale-up the number of such centers in the near future.

These guidelines are part of the ongoing effort to rapidly scale-up antiretroviral therapy (ART) throughout the country. A lot of effort has gone into the preparation of these guidelines. We have been able to incorporate the latest guidance from WHO on Antiretroviral therapy and PMTCT including lessons learnt from our own in country experience with ART over the last 8 years, when the first edition of the guidelines came out.

These guidelines are meant for use by doctors, nurses and paramedics who are actively involved in care of the patients. I would like to acknowledge the Technical Working Group for the incessant effort they have put in bringing out these guidelines. I would also like to thank our partner organizations- the WHO, UNDP, and FHI 360 as well as all the other individuals and organizations for their time and effort in bringing out these guidelines. A special thank you goes to Dr Hemant Ojha, Dr. Sushil Shakya, Dr. Sashi Sharma, Dr. Prem Khadga, Dr. Lisa Stevens, and Dr. Atul Dahal who were instrumental in ensuring the guidelines capture the latest development and experiences in regards to HIV treatment and care.

I am sure this revised guideline will enable doctors and nurses to deliver a high standard of HIV treatment and care services, which will improve the quality of life for people living with HIV/AIDS in Nepal.

Dr. Krishna Kumar Rai
Director

National Center for AIDS and STD Control

Abbreviations

**3TC**	Lamivudine
**ABC**	Abacavir
**AFB**	Acid fast bacillus
**AIDS**	Acquired immune deficiency syndrome
**ALT**	Alanine aminotransferase (also known as SGPT)
**ANC**	Absolute neutrophil count
**ARV**	Anti-retroviral
**ART**	Anti-retroviral therapy
**ATT**	Anti-tuberculosis therapy
**ATV**	Atazanvir
**AZT**	Azidothymidine (zidovudine)
**bPI**	Boosted protease inhibitor
**CBC**	Complete blood count
**CD**	Cluster of differentiation
**CNS**	Central nervous system
**CXR**	Chest x-ray
**d4T**	Stavudine
**DBS**	Dried blood spots
**DC**	Differential leucocyte count
**ddI**	Didanosine
**ECP**	Emergency contraceptive pill
**EFV**	Efavirenz
**ELISA**	Enzyme linked immunosorbent assay

**FI**	Fusion inhibitor
**fosAPV**	Fosamprenavir
**FTC**	Emtricitabine
**GI**	Gastrointestinal
**Hb**	Hemoglobin
**HBV**	Hepatitis B virus
**HCV**	Hepatitis C virus
**HIV**	Human immunodeficiency virus
**HIV-DR**	HIV drug resistance
**HTC**	HIV Testing & Counselling
**IDU**	Injecting drug user
**INSTI**	Integrase strand transfer inhibitor
**IPT**	Isoniazid preventive therapy
**IRIS**	Immune reconstitution inflammatory syndrome
**IUD**	Intrauterine device
**LFT**	Liver function test
**LPV**	Lopinavir
**LPV/r**	Lopinavir boosted with ritonavir
**MTCT**	Mother-to child transmission (of HIV)
**NNRTI**	Non-nucleoside reverse transcriptase inhibitor
**NRTI**	Nucleoside reverse transcriptase inhibitor
**NsRTI**	Nucleoside analog reverse transcriptase inhibitor
**NtRTI**	Nucleotide analog reverse transcriptase inhibitor
**NVP**	Nevirapine
**OCP**	Oral contraceptive pill
**OI**	Opportunistic infection
**OST**	Oral substitution therapy
**PEP**	Post exposure prophylaxis

**PCP**	Pneumocystis jiroveci pneumonia
**PCR**	Polymerase chain reaction
**PI**	Protease Inhibitor
**PLHIV**	People living with HIV
**PMTCT**	Prevention of Mother to Child Transmission
**PTB**	Pulmonary TB
**/r**	Low dose ritonavir
**RTV**	Ritonavir
**sdNVP**	Single dose nevirapine
**SGPT**	Serum glutamic pyruvic transaminase (also known as ALT).
**SQV**	Saquinavir
**RT**	Reverse transcriptase
**STI**	Sexually transmitted infections
**TB**	Tuberculosis
**TDF**	Tenofovir disoproxil fumarate
**TC**	Total leucocyte count
**VCT**	Voluntary counseling and testing (of HIV)
**VL**	Viral load
**WHO**	World health organization
**ZDV**	Zidovudine, (Also known as AZT)

TABLE OF CONTENTS

I.	Background	1
II.	Principles of Antiretroviral Therapy	2
III.	Indications for Antiretroviral Therapy	4
IV.	Antiretroviral Drugs and their actions	7
V.	Choice of Antiretroviral Regimen	14
VI.	Monitoring	21
VII.	Changing Antiretroviral Drugs	26
VIII.	Monitoring of HIV drug resistance	31
IX.	Discontinuation or Interruption of Antiretroviral Therapy	32
X.	Antiretroviral Therapy in Pregnancy	33
XI.	Tuberculosis and Antiretroviral Therapy	41
XII.	Hepatitis C Co-infection and ART	45
XIII.	Hepatitis B Co-infection and ART	45
XIV.	Immune Reconstitution Inflammatory Syndrome (IRIS)	46
XV.	Cotrimoxazole Prophylaxis in Adults	46
XVI.	Post Exposure Prophylaxis (PEP) of HIV	49
XVII.	Management of HIV in Infants and Children	56
XVIII.	Antiretroviral Therapy in Injecting Drug Users	61
XIX.	Annexes	65

I. Background

The first case of AIDS was reported in 1981 in the USA. Since then, AIDS has become the most devastating and threatening disease of humans. More than 60 million people have been infected and about 34 million people are estimated to be currently living with HIV, among whom one-third are between 15 and 24 years of age. Initially, the epidemic was concentrated in the African continent. But it continues to spread in Asia, particularly in South Asia. In South and South East Asia, there are approximately 4 million people living with HIV. In Nepal, the first case of HIV was diagnosed in 1988. The major mode of transmission in Nepal is heterosexual. The available data show that there is a high prevalence of HIV in high-risk groups such as injecting drug users and female sex workers. Currently, it is estimated that there are approximately 50,200 people living with HIV (PLHIV) in Nepal, with an estimated 4,906 deaths in 2010.

In 1986, antiretroviral therapy was initially introduced in other countries with the first drug Zidovudine (ZDV). Over the next few years, other antiretroviral drugs (NRTIs, NNRTIs and PIs) were introduced. Initially, mono and dual therapies were used but the problem of resistance emerged. At present, 3 or more ARV drugs are recommended worldwide for the treatment of people with HIV infection. Since the use of combination therapy, this disease has been transformed into a chronic condition. Although the use of antiretroviral therapy is not the final answer to HIV prevention and care programs, treatment and prevention have been proven to be inextricably linked. ART dramatically decreases the chance of transmitting the virus both sexually and from mother-to-child. The delivery of effective care and antiretroviral treatment for people living with HIV in the poorest countries is considered an urgent priority and is now an integral part of HIV transmission prevention programs. Initially, antiretroviral therapy was very expensive and so, unaffordable in most developing countries. As drugs are increasingly affordable, the development of guidelines on the appropriate and rational use of ART has become relevant in developing countries.

These current guidelines are intended basically for use by medical practitioners who prescribe ARV therapy to the people infected with HIV. Guidelines for the treatment and management of HIV infection have been produced in a number of countries of Europe, USA, Australia, India, Thailand, etc. and by WHO. While these guidelines attempt to represent the current state of knowledge, it is inevitable that as HIV is a rapidly evolving medical field new information will change therapeutic choices and preferences. This should be kept in mind while using these guidelines.

II. Principles of Antiretroviral Therapy

Goals of Antiretroviral Therapy

- Maximal and durable suppression of viral load
- Restoration and/or preservation of immunologic function
- Reduction of HIV-related morbidity and mortality
- Improvement of quality of life of HIV infected persons
- Prevention of Mother to Child Transmission (PMTCT)
- Post Exposure Prophylaxis (PEP)

Preconditions for Starting Antiretroviral Therapy

The following specific facilities and services are desirable before starting Antiretroviral Therapy

- Availability of HIV testing and counseling (HTC) services along with follow-up counseling services.
- Medical services capable of managing common HIV-related infections including opportunistic infections (OIs) and sexually transmitted infections (STIs).
- Routine laboratory services, preferably with access to CD4 lymphocyte count and PCR facility for viral load count. Lack of viral load testing and even CD4 testing should not preclude initiation of ART.
- Access to antiretroviral drugs and other drugs to treat OIs and other associated diseases.

It is recommended that medical doctors who are trained in clinical management of HIV initiate and supervise antiretroviral therapy.

Evaluation of Patients before Starting Antiretroviral Therapy

A detailed evaluation is essential prior to initiating antiretroviral therapy and should aim to:

- Assess the clinical stage of HIV infection.
- Identify past HIV-related illnesses.

- Identify current HIV-related illnesses that will require treatment.
- Identify co-existing medical conditions and treatments that may influence the choice of therapy.

Before Initiating Therapy, The Following Evaluations should be Performed:

- A history including past illnesses with emphasis on OIs and other conditions
- Psychological and psychiatric history.
- Assessment of family and social support
- Practices regarding safe sex and injecting drug use
- Physical examination

Physical Examination and Lab Tests before Initiating ARV Therapy:

Body weight of the patient

Skin: look for Herpes Zoster, HIV dermatitis and other skins conditions.

Lymphadenopathy.

Oropharyngeal mucosa: look for candidiasis, and hairy leucoplakia, Kaposi sarcoma.

Examination of abdomen, heart, lungs, neurological, musculoskeletal and genitourinary systems.

Laboratory Tests:

TC, DC, Hb%

ALT/SGPT- If needed LFT (Liver function test)

Serum creatinine- If needed Kidney function test (Urea, Electrolytes)

Blood sugar level

Chest X ray, Sputum for AFB

Hepatitis B and Hepatitis C

Urine pregnancy test as indicated in female

Urinalysis to assess for proteinuria

CD4 cell count

For women, cervical pap smear or other method of cervical cancer screening, if available.

III. Indications for Antiretroviral Therapy

Recommendations for initiating antiretroviral therapy in adults and adolescents with documented HIV infection are given in the box below.

If CD4 Testing Available:

It is recommended to document baseline CD4 counts and to offer ART to patients with:

WHO Stage 4 disease, irrespective of CD4 cell count

WHO Stage 3 disease, irrespective of CD4 cell count

WHO Stage 1 or 2 disease with CD4 cell counts less than 350/mm^3

If CD4 Testing Unavailable:

It is recommended to offer ART to patients with:

WHO Stage 4 disease

WHO Stage 3 disease

Note: WHO Stage 1 and 2, Treatment is Not Recommended When CD4 Testing is Not Available

Start ART in those with chronic active Hepatitis B irrespective of CD4 count or WHO Clinical Stage

Note: In co-infection with other diseases, treatment of tuberculosis and some other opportunistic infections the priority is usually to start OI treatment before antiretroviral therapy. However, recent evidence suggests that ART should be started early in the setting of acute AIDS-related OIs, if there are no major contraindications to doing so. Waiting to complete OI treatment before initiating ART appears to be associated with a higher risk of AIDS-related disease progression and/or death without any significant benefit in terms of safety or virological response.

All people living with HIV (PLHIV) with TB should start TB treatment first and then begin ART as soon as possible, but not more than 8 weeks after ATT.

Note: Pregnant and breastfeeding postpartum women should start ART

Those eligible for ART as above should begin life-long therapy as soon as possible.

Those not yet eligible should start triple ARVs as short term prevention from the 14th week of pregnancy onwards and continue until 1 week after cessation of breastfeeding. See Nepal PMTCT Guidelines for details.

WHO Clinical Staging of HIV/AIDS for Adults and Adolescents

Clinical Stage 1

- Asymptomatic
- Persistent generalized lymphadenopathy

Clinical Stage 2

- Unexplained moderate weight loss (<10% of presumed or measured body weight)[a]
- Recurrent respiratory tract infections (sinusitis, tonsillitis, otitis media and pharyngitis)
- Herpes zoster
- Angular chelitis
- Recurrent oral ulceration
- Papular pruritic eruptions
- Seborrhoeic dermatitis
- Fungal nail infections

Clinical Stage 3[b]

- Unexplained severe weight loss (>10% of presumed or measured body weight)
- Unexplained chronic diarrhea for longer than one month
- Unexplained persistent fever (above 37.5°C intermittent or constant, for longer than 1 month)
- Persistent oral candidiasis
- Oral hairy leukoplakia
- Pulmonary tuberculosis
- Severe bacterial infections (such as pneumonia, empyema, pyomyositis, bone or joint infection, meningitis or bacteraemia)
- Acute necrotizing ulcerative stomatitis, gingivitis or periodontitis
- Unexplained anaemia (<8g/dl), neutropaenia (<0.5 × 109 per litre) and/or chronic thrombocytopaenia (<50 × 109 per litre)

Clinical Stage 4[c]

- HIV wasting syndrome
- Pneumocystis pneumonia
- Recurrent severe bacterial pneumonia
- Chronic herpes simplex infection (orolabial, genital or anorectal of more than one month's duration or visceral at any site)
- Oesophageal candidiasis (or candidiasis of trachea, bronchi or lungs)
- Extrapulmonary tuberculosis
- Kaposi sarcoma
- Cytomegalovirus infection (retinitis or infection of other organs)
- Central nervous system toxoplasmosis
- HIV encephalopathy
- Extrapulmonary cryptococcosis including meningitis
- Disseminated non-tuberculous mycobacterial infection
- Progressive multifocal leukoencephalopathy
- Chronic cryptosporidiosis
- Chronic isosporiasis
- Disseminated mycosis (extrapulmonary histoplasmosis or coccidiomycosis)
- Recurrent septicaemia (including non-typhoidal Salmonella)
- Lymphoma (cerebral or B-cell non-Hodgkins)
- Invasive cervical carcinomas
- Atypical disseminated leishmaniasis
- Symptomatic HIV-associated.nephropathy or symptomatic HIV-associated cardiomyopathy

a. Assessment of body weight in pregnant woman needs to consider the expected weight gain of pregnancy.

b. Unexplained refers to where the condition is not explained by other causes.

c. Some additional specific conditions can also be included in regional classifications (such as penicilliosis in Asia).

IV. Antiretroviral Drugs and Their Actions

Categories of Approved Antiretroviral Drugs Include The Following:

1. A. Nucleoside analog reverse transcriptase inhibitors (NsRTI)

   B. Nucleotide analog reverse transcriptase inhibitors (NtRTI)
2. Non-nucleoside analog reverse transcriptase inhibitors (NNRTIs)
3. Protease inhibitors (PIs)
4. New class of drugs: Fusion inhibitors (FI), CCR5 Antagonists, Integrase Strand Transfer Inhibitors (INSTI)

MECHANISM AND SITE OF ACTION OF ANTIRETROVIRAL AGENTS

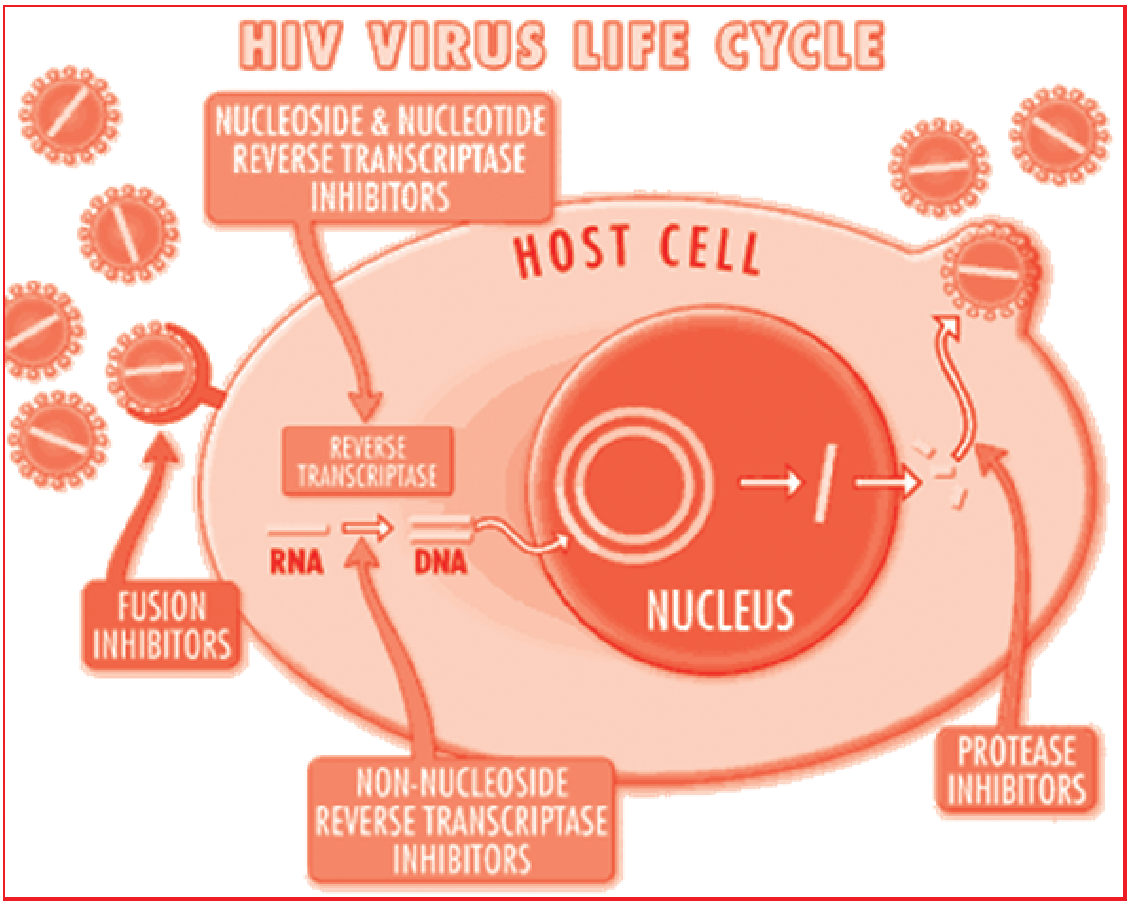

The first effective class of antiretroviral drugs was the **Nucleoside Analogues.** They act by incorporating themselves into the DNA of the virus, thereby stopping the building process. The resulting DNA is incomplete and cannot create new virus. **Nucleotide Analogues** work in the same way as nucleosides, but they have a nonpeptidic chemical structure.

Non-nucleoside Reverse Transcriptase Inhibitors (NNRTIs) stop HIV production by binding onto reverse transcriptase and preventing the conversion of RNA to DNA. These drugs are called "non-nucleoside" inhibitors because even though they work at the same stage as nucleoside analogues, they are not nucleoside analogues.

Protease Inhibitors work at the last stage of the viral reproduction cycle. They prevent HIV from being successfully assembled and released from the infected CD4 cell. **Fusion Inhibitors** work to prevent fusion and entry of the virus to the target cell (CD4). Integrase Inhibitors work to prevent the integration of the HIV proviral DNA into the human DNA. **CCR5 Antagonists** work by blocking co-receptors needed for the virus to enter the cell.

Table 1: Nucleoside/Nucleotide Reverse Transcriptase Inhibitors (NsRTIs/NtRTIs)

Generic Name	Dose	Food Effect	Adverse Effects	Interaction with Other Antiretroviral Therapy
Abacavir (ABC)	300 mg twice daily or 600mg OD	Take without regards to meal	Hypersensitivity reaction in 3 to 5% (can be fatal), fever, rash, fatigue, nausea, vomiting, anorexia, respiratory symptoms (sore throat, cough, shortness of breath)	Rechallenge after reaction can be fatal. Some studies show that ABC has been associated with increased cardiovascular risk; these drugs should be used with caution in high risk patients. This was not substantiated in other studies
Didanosine (ddI)	Weight < 60kg: 250mg once daily<br>Weight ≤ 60kg: 400mg once daily;	Take on empty stomach (1/2 hour before or 2 hours after meal)	Pancreatitis Peripheral neuropathy Lactic acidosis with hepatic steatosis (rare but potentially life-threatening toxicity) Potential asso-ciation with noncirrhotic portalhypertension	Avoid TDF and ddI concomitant use. Try to avoid use with d4T due to increased toxicity. Recent studies show that ddI has been associated with increased cardiovascular risk; these drugs should be used with caution in high risk patients.
Lamivudine (3TC)	150 mg twice daily<br>Or 300 mg once daily	Take without regards to meals	Minimal toxicity	Do not coadminister with FTC. Severe acute exacerbation of hepatitis may occur in HBV coinfected patients who discontinue 3TC.
Stavudine (d4T)	30 mg twice daily	Take without regards to meals	Pancreatitis, peripheral neuropathy, lipoatrophy, lactic acidosis with hepatic steatosis, hyperlipidemia, rapidly progressive ascending neuromuscular weakness (rare)	Antagonist with ZDV Try not to use with ddI due to increased toxicity

Generic Name	Dose	Food Effect	Adverse Effects	Interaction with Other Antiretroviral Therapy
Zidov-udine (ZDV, AZT)	300 mg twice daily	Take without regards to meals	Anaemia, neutropenia, bone marrow suppression, gastrointestinal intolerance, headache, insomnia, myopathy, lactic acidosis, skin & nail hyperpigmentation.	Antagonist with d4T
Tenofovir (TDF) NtRTI	300 mg OD	Take without regards to meal	Asthenia, headache, diarrhea, nausea, vomiting, and flatulence Renal insufficiency, Fanconi syndrome. Dosage adjustment in renal insufficiency recommended Osteomalacia Potential for decrease in bone mineral density Severe acute exacerbation of hepatitis may occur in HBV-coinfected patients who discontinue TDF.	Avoid TDF and ddI concomitant use. Serum creatinine and urinalysis for proteinuria should be monitored at baseline and follow-up (every 6 months). If reduced creatinine clearance to <50 reduce dose: Cr Cl 30-49 TDF 300mg q 48 hrs Cr Cl 10-29 or on dialysis TDF 300mg q 72-96 hrs.
Emtri-citabine (FTC)	200mg OD	Take without regard to meal		Do not coadminister with 3TC. Severe acute exacerbation of hepatitis may occur in HBV-coinfected patients who discontinue FTC.

"Class side effect": All nucleoside analogs have been associated with lactic acidosis and hepatic steatosis.

Table 2: Non-nucleoside Reverse Transcriptase Inhibitors (NNRTIs)

Generic Name	Dose	Food effects	Adverse Effect
Nevir-apine (NVP)	200 mg once daily for 14 days followed by 200 mg twice daily. If patient is on rifampicin, start bid dose with no dose escalation at 2 weeks. If switching from EFV to NVP, start bid. No dose escalation is needed.	Take without regards to meals	Hepatitis (usually within 12 wks), life-threatening hepatic toxicity Contraindicated as initial therapy for women if CD4 count is >250 due to increased hepatotoxicity in this group Skin rash occasionally progressing to severe conditions including TEN and Stevens Johnson syndrome.
Efavirenz (EFV)	600 mg once daily (bed time administration is suggested to decrease CNS side effects)	Avoid taking after high fat meals	CNS symptoms (dizziness, somnolence, insomnia, confusion, hallucinations, agitation), teratogenicity, and personality change. Rash occurs, but less common than NVP

- Patients who develop hepatic toxicity while treated with Nevirapine should not be restarted on that drug.
- Efavirenz is contraindicated in the first trimester of pregnancy.

Table 3: Protease Inhibitors (PIs)

Generic Name	Dose	Food Effect	Adverse Effects
Lopinavir/ ritonavir (LPV/r) Heat stable tablets	200mg Lopinavir/ 50mg Ritonavir Fixed dose tablets 2 tablets twice daily	with or without food	Diarrhea, nausea, vomiting, abnormal lipid profiles, glucose intolerance
Saquinavir/ ritonavr (SQV/r) Requires refrigeration Less potent than other PIs.	1000mg saquinavir + 100 mg ritonavir twice daily	No food effect when taken with ritonavir	Diarrhea, nausea, vomiting, headache, photosensitivity
Atazanavir/ ritonavir (ATV/r) Requires refrigeration, but heat-stable form in development	300mg Atazanavir + 100mg ritonavir once daily	Take with food	Hyperbilirubinemia. Less lipid problems than LPV/r Hyperglycemia, fat maldistribution, nephrolithiasis Interaction with acid blocking agents. Dosing changes when given with acid-blockers
Indinavir/ ritonavir (IDV/r)	800 mg Indinavir/ 100mg ritonavir twice daily	Take with food	Nephrolithiasis, increase indirect bilirubin, headache. Rash, diarrhea, nausea, mood disorder, altered taste. Less well tolerated. Not preferred in hot climates.

"Class side effect": All PIs can produce increased bleeding in hemophilia, GI intolerance, altered taste, increased liver function test and bone disorder, and all have been associated with metabolic abnormalities, such as hyperglycemia, insulin resistance, and increase in triglycerides, cholesterol and body fat distribution (lipodystrophy).

Table 4: Management of Serious Adverse Effects of Antiretroviral Drugs

Adverse Effect	Major ARVs	Recommendations
Acute pancreatitis	d4T and ddI	Discontinue ART. Give supportive treatment with laboratory monitoring. Resume ART with an NRTI with low pancreatic toxicity risk such as AZT or TDF.
Diarrhoea	ddI (buffered formulation), LPV/r and SQV/r	Usually self-limited, without need to discontinue ART. Symptomatic treatment should be offered.
Drug eruptions (mild to severe, including Stevens-Johnson syndrome or toxic epidermal necrolysis)	NVP, EFV (rarely)	In very mild cases, symptomatic care. EFV rash often stops spontaneously after 3-5 days without need to change ARVs. If moderate rash, non-progressing and without mucosal involvement or systemic signs, consider a single NNRTI substitution (i.e. from NVP to EFV). In moderate and severe cases, discontinue ART and give supportive treatment. After resolution, resume ART with LPV/r based regimen or triple NRTI if no other choice.
Dyslipidaemia, insulin resistance and hyperglycaemia	PIs	Consider replacing the suspected PI by drugs with less risk of metabolic toxicity. Adequate diet, physical exercise and antilipaemic drugs should be considered.
GI intolerance, with taste changes, nausea, vomiting, abdominal pain and diarrhoea.	All ARVs (less frequent with d4T, 3TC, FTC and ABC)	Usually self-limited, without need to discontinue ART. Symptomatic treatment should be offered.
Anaemia and Neutropenia	AZT	If severe (Hg <7 g% and/or ANC <750 cells/mm^3), replace by an ARV with minimal or no bone marrow toxicity (e.g. TDF) and consider blood transfusion.

Adverse Effect	Major ARVs	Recommendations
Hepatitis	All ARVs (particularly with NVP and ritonavir-boosted PIs)	If ALT is at more than five times baseline, discontinue ART and monitor. After resolution, restart ART and replace the drug most likely associated with the condition (e.g. EFV replaces NVP).
Hyperbilirubinaemia (indirect)	ATV	Generally asymptomatic but can cause scleral icterus (without ALT elevations). Replace ATV with other PI.
Hypersensitivity reaction with respiratory symptoms, fever and without mucosal involvement.	ABC	Discontinue ABC and do not restart. Symptomatic treatment. Re-exposure may lead to a severe and potentially life-threatening reaction.
Lactic acidosis	All NRTIs (particularly d4T and ddI)	Discontinue ART and give supportive treatment. After clinical resolution, resume ART with TDF.
Lipoatrophy and lipodystrophy	All NRTIs (particularly d4T)	Early replacement of the suspected ARV drug (e.g. d4T replaced with TDF or ZDV).
Neuropsychiatric changes (sleep disturbances; depression; behavioural, concentration and personality changes)	EFV	Usually self-limited, without the need to discontinue ART. If intolerable to patient, replace with NVP or LPV/r. Single substitution recommended without stopping ART.
Renal toxicity (renal tubular dysfunction)	TDF	Consider substitution with AZT
Peripheral neuropathy	d4T and ddI	Replacement by AZT or TDF. Symptomatic treatment (amitriptyline, Vitamin B6)

V. Choice of Antiretroviral Regimen

The Choice of Regimen Depends on

- Cost of therapy
- Availability
- Affordability of drugs
- Convenience and likelihood of adherence.
- Regimen potency, tolerability and adverse effect profile
- Possible drug interactions and potential for alternate treatment options in the event that the initial drug regimen fails.

Antiretroviral therapy with dual drug regimen is not recommended except for in some cases of post exposure prophylaxis of HIV.

The combination of a either an NNRTI or a protease inhibitor with 2 NRTIs is potent and causes durable suppression of viral replication. Combination of ritonavir with another PI results in a boosting effect by increasing plasma concentration of these drugs, thereby reducing their doses frequency and pill burden. Currently, several regimens with acceptable antiviral potency are available. These regimes are composed of three or four drugs. Two NsRTIs generally form the backbone of most combinations (see box below).

Principle of Combination:

1. 2 NRTI + NNRTI

or

2. 2 NRTI + PI

or

3. 2 NRTI + 1 NRTI (Abacavir)

Note: Triple NRTI therapy can be ZDV+3TC+ABC or ZDV+3TC+TDF; do not use other triple NRTI options

Possible NRTI Combination Include:

Zidovudine + Lamivudine (ZDV/3TC)

Tenofovir + Lamivudine (TDF/3TC)

Zidovudine + Didanosine (ZDV/ddI)

Didanosine + Lamivudine (ddI/3TC)

Abacavir + Lamivudine (ABC/3TC)

ZDV and d4T should not be used together due to their antagonistic effects.

Didanosine and Stavudine should not be used together due to high risk of toxicity.

Didanosine and Tenofovir should not be used to together due to poor efficacy and side effects.

Some patients will have started Stavudine + Lamivudine (d4T/3TC) in the past, this should no longer be used as a first line regimen for new patients as it is "phased-out" of Nepal due to high risk of toxicity of stavudine.

The common recommended regimens are further given in table 5.

Table 5: First-line ARV Regimens in Adults and Adolescents

ARV Regimen	Major Potential Toxicities	Usage in Women of Childbearing Age or Pregnant	Usage in TB Co-infection[a]
ZDV/3TC/NVP	ZDV related GI intolerance, anaemia, and neutropenia, NVP related hepatoxicity and severe rash	Yes	Use with caution in rifampicin based regimens[a]
TDF/3TC/NVP	Tenofovir related renal insufficiency, Fanconi syndrome. HBV exacerbation on discontinuation. NVP related hepatoxicity & severe rash	Yes	Use with caution in rifampicin based regimens[a]
ZDV/3TC/EFV	ZDV related GI intolerance, anaemia & neutropenia, EFV related CNS toxicity & potential for teratogenicity	Only during 2nd and 3rd trimester. EFV should not be given to women in the first trimester of pregnancy or women of childbearing potential, unless effective contraception can be assured	Yes
TDF/3TC/EFV	Tenofovir related renal insufficiency, Fanconi syndrome. HBV exacerarbation on discontinuation. EFV related CNS toxicity & potential for teratogenicity	Only during 2nd and 3rd trimester. EFV should not be given to women in the first trimester of pregnancy or women of childbearing potential, unless effective contraception can be assured	Yes

If patient is on ART containing stavudine (d4T), they should be changed from stavudine to either zidovudine or tenofovir.

New patients should not be started on stavudine (d4T) containing regimens.

[a]See Section (Tuberculosis and Antiretroviral Therapy).
[b]See Section (Antiretroviral Therapy in Pregnancy and breast feeding).

What to Use for Confirmed HIV 2 Cases

For HIV-2 infections, the triple NRTI or PI based regimens should be used because of inherent resistance of HIV 2 to NNRTI drugs. PI based regimen is the preferred option. (ie. ZDV+3TC+LPV/r)

When to Start with Tenofovir (TDF) Instead of Zidovudine (ZDV):

- ZDV is preferred in most cases
- TDF should be used in the case of anemia or if ZDV is otherwise not tolerated.
- TDF does not require hemoglobin monitoring.
- ZDV may cause nausea, headache, anemia and neutropenia.
- WHO has recommended that Stavudine (d4T) be "phased out" due to unacceptable rates of toxicity.
- Patients started or switched to Stavudine (d4T) for anemia, can switch to Zidovudine after 6-12 months of stable Hb above threshold, to avoid d4T toxicities. If unable to tolerate ZDV, switch to Tenofovir.
- Abacavir can be used in the place of Tenofovir.

Some Other Considerations for NRTIs:

- Do not combine "d-drugs" (ddI, d4T).
- Do not give single d-drugs with pre-existing neuropathy.
- Do not combine 3TC and FTC.
- Do not combine ddI and TDF.

The Choice between Nevirapine (NVP) and Efavirenz (EFV):

- NVP and EFV are both potent NNRTIs.
- The major toxicities associated with EFV are central nervous system (CNS) related, metabolic toxicity, teratogenicity and rash. The CNS symptoms typically abate within the first month of therapy.
- NVP has higher incidence of rash, which may be severe and life threatening. NVP has also a higher risk of hepatotoxicity.

Thus for most patients ZDV/3TC/NVP will be preferred as a first line ART. In patients with Hb less than 7gm% the regimen of choice should be TDF/3TC/NVP. In a female patient who is in the first trimester of pregnancy or likely to be pregnant, EFV should be avoided. In a patient with a certain side effects, an alternative drug should be used.

Triple NRTI-based First-line Regimens

Triple NRTI-based first-line regimens such as ZDV+3TC+ABC or ZDV+3TC+TDF can be recommended in specific circumstances where both NNRTIs are contraindicated or not tolerated. Other triple NRTI combinations should not be used. Triple NRTIs have the advantage that they still preserve the PI and NNRTI class for second-line ART. These regimens can be used in the following circumstances:

- Intolerance or resistance to NNRTIs;
- Psychiatric disorders;
- Women starting ART with CD4 >250, since great risk of NVP toxicity.
- Pre-existing liver disease- an increase of the ALT level by more than 3-5 fold and established cirrhosis;
- Coinfection with HBV or HCV;
- HIV-2 infection due to intrinsic resistance to NNRTI class; and
- Cotreatment of TB in women of child-bearing age and where adequate contraception cannot be guaranteed, and when NVP and boosted PIs cannot be used.

ZDV+3TC+ABC has short-term inferior virological efficacy at least in patients with high initial viral loads, but comparative immunological efficacy to ZDV+3TC+EFV regimen (CD4 recovery). ZDV+3TC+TDF is now also recommended by WHO. Triple NRTI regimens may be used especially in the management of tuberculosis and HIV co-infection; management of hepatitis C and HIV co-infection; and management of hepatitis B and HIV co-infection. Other triple NRTI-based regimens, such as ZDV+TDF+ABC or TDF+3TC+ddI have unacceptably high virological failure rates and high incidence of the K65R mutation and should not be used.

PI Based Regimen in First Line

Boosted PIs are usually reserved for second-line ART. They can exceptionally be used as part of first-line ART in combination with two NRTIs when triple NRTI regimen is not available or deemed inappropriate and when there are contraindications to NNRTIs (i.e. neither EFV nor NVP can be prescribed) including:

- Psychiatric disorders;
- An increase of the ALT level by more than 3-5 fold;
- Cirrhosis;
- Pregnancy with CD4 count of 250-350 cells/mm^3, particularly in the 1st trimester of pregnancy (as EFV is contraindicated);
- HIV-2 infection due to intrinsic resistance to NNRTI class
- A baby under two years of age with previous exposure to PMTCT NNRTIs.
- A woman starting ART within 12 months of receiving PMTCT single-dose NVP without an NRTI tail;

If a first-line ART regimen containing a PI fails, there are very limited options for subsequent regimens within the public sector in Nepal. In general therefore, it is recommended that PIs be reserved for second-line ART.

Table 6: ART Regimens Recommended for Women with Prior Exposure to PMTCT Regimen

Previous ARV Exposure for PMTCT	Regimen to Use at Later Initiation of ART
sdNVP (+/- antepartum AZT) with NO AZT/3TC tail in last 12 months	Start PI based regimen (less preferred is triple NRTI)
sdNVP (+/- antepartum AZT) with AZT/3TC tail in last 12 months	Start NNRTI regimen*
sdNVP (+/- antepartum AZT) more than 12 months ago (with or without tail)	Start NNRTI regimen*
Triple ARV- prophylaxis	Start NNRTI regimen*

*If started on NNRTI regimen and previous sd-NVP (or triple ARV with EFV and no tail), try to check viral load at 6 months. If >5,000 switch to second-line ART with PI.

VI. Monitoring

Follow-Up Schedule

Clinical and Adherence Monitoring Visits:

Once ART is started, follow up schedule should be as follows:

First month: two visits (every 2 weeks)

Second + Third month: every month

Fourth month onwards: one visit every three months

More frequent visits will be scheduled, if the patient develops symptoms or experiences difficulties in adhering to the medications.

Laboratory Monitoring:

First Year

2nd week
CBC and Liver Function tests (ALT if on NVP)

Month 1
CBC
LFT if on NVP
Other necessary investigation if and as required

Month 2
CBC
LFT if on NVP
Other necessary investigation if and as required

Month 3
CBC
LFT if on NVP
Other necessary investigation if and as required

Month 6
CBC, platelets
LFTs
CD4 Cell Count
If on Tenofovir, creatinine and urinalysis for proteinuria

Month 9
CBC and LFT

Month-12
CBC
LFT
CD4 Cell count
Other tests as needed

Subsequent Years:

Quarterly
CBC

Every 6 Months
CD4 Cell count
LFT
If on Tenofovir, creatinine and urinalysis for proteinuria.
Other tests as needed

Yearly
Fasting lipids and blood glucose for those on protease inhibitors

Viral Load Testing for Those on ART:

For a patient who is not responding to treatment, a viral load test will be requested whenever feasible.

- Where available, obtain viral load to confirm treatment failure.
- If resources allow, obtain viral load every 6 months to detect viral replication.
- Additional pediatric recommendation: Babies under 12 months starting NVP-based ART after NNRTI exposure through maternal or infant PMTCT prophylaxis, should have viral load checked 6 months after start, if possible.
- Pregnant women at near term (36 weeks gestation) who are on ART and considering an elective caesarian section, should be offered viral load testing if possible.
- Women starting NNRTI based ART after PMTCT exposure to sd-NVP or triple ARV with EFV and no ZDV/3TC tail, should have viral load testing 6 months after start, if possible.

A Persistent Viral Load >5,000 Confirms Failure.

Adherence

A high degree of adherence to ARV drugs is necessary for optimal virological suppression. Studies indicate that >95% of the doses should be taken for optimal suppression. Lesser degree of adherence is more often associated with virological failure. Adherence should be assured before initiation of antiretroviral therapy. The patient should fully understand the importance of adherence to antiretroviral therapy. Adherence counseling and patient education should be done at every follow-up visit.

Promoting Adherence: Issues to Consider

- Health care personnel should be supportive and non-judgmental
- Simplified treatment regimen e.g. once or twice a day dosing, less number of pills with minimal side effects.
- The motivation of the individual to begin and continue therapy
- The individual's understanding of the importance of adherence and its relationship to drug resistance
- The impact of therapy on the individual's lifestyle and psychological well being
- Education of patient's family and friends.
- The provision of memory aids (such as pill boxes, bleepers, medication record cards, mobile phone messages), to establish and maintain a pill taking routine
- Treatment of any underlying mental health problems
- Management of side effects
- The potential risks and benefits of therapy, in the short and long term.
- The provision of written information to provide support outside the clinic setting, covering the regimen, potential side effects and the consequences of low adherence.

Advice to Patients for Missed ARV Doses:

- When you notice that you missed a dose, take your pill right away.

For The NEXT DOSE

- If the next planned pill-taking time is four hours away or less, DO NOT take your next dose. Instead wait four hours and then take your next dose. After this follow your regular dosing schedule.
- Do not take two doses at one time.
- If is it already time for the next dose, just take that dose and carry on with the treatment schedule.

Adherence Performance Chart for Nepal

Table 7: Adherence Based on Number of Pills Prescribed Per Day

# Pills in the regimen	% Adherence		
	> 95%	80-95%	<80%
1 Pill per day (e.g. TDF/3TC/EFV FDC)	1 or less	2 to 6	7 or more
2 Pills per day (e.g. ZDV/3TC/NVP FDC)	3 or less	4 to 12	13 or more
3 Pills per day (e.g. ZDV/3TC + EFV)	4 or less	5 to 18	19 or more
4 Pills per day	6 or less	7 to 24	25 or more
5 Pills per day	8 or less	9 to 30	31 or more
6 Pills per day (e.g. ZDV/3TC + LPV/r)	9 or less	10 to 36	37 or more
7 Pills per day (e.g. TDF + 3TC + LPV/r)	10 or less	11 to 42	43 or more

Criteria for Treatment Success

Table 8: Criteria for Treatment Success

Criteria	Virological	Immunological	Clinical
**Marker**	Viral load	CD4 cell count	Clinical stage
**Time**	6 months	6 months	By 12 weeks of treatment initiation should be asymptomatic or have few symptoms.
**Suggested Ranges**	<5,000 copies/ml Ideally, this should be undetectable.	Increase from baseline by at least 50-100 cells/mm^3	Treatment Stage 1 or 2

Toxicity and Failure of Antiretroviral Therapy

Appearance of HIV related opportunistic infections may mean failure of the antiretroviral regimen. Detailed history taking and physical examination should be done at least every 3 months. At the time of follow-up visits, monitoring should be done for CBC, serum ALT, serum creatinine, blood glucose and serum lipids depending on the drug regimen and possible drug adverse effects. Whenever feasible, CD4 count should be performed at least every 6 months. In patients with optimal antiretroviral therapy CD4 counts increase by >100 cells/mm^3 in the first 6-12 months in ARV naive, adherent patient with drug susceptible virus. Immunologic failure is indicated by a fall in CD4 counts higher than 50% from the peak value or a return to, or below, the pre-therapy baseline, or by persistent CD4 <100 cells/mm^3. Viral load is available on a limited basis in Nepal. Ideally, viral load will be checked starting at 6 months after ART initiation and rechecked every 6 months (at least once a year). In addition, it should be checked after a history of non-adherence and at the time of suspected clinical or immunologic failure to confirm the presence or absence of virologic failure.

VII. Changing Antiretroviral Drugs

The usual reasons for changing antiretroviral drug regimen include:

- Drug adverse effects
- Inconvenient regimens (dosing and number of pills that may compromise adherence).
- Treatment failure
- Occurrence of active tuberculosis and pregnancy.
- Concomitant illness (i.e. Hepatitis B)

Table 9. Toxicities of First-line ARVs and Recommended Drug Substitutions

ARV DRUG	COMMON ASSOCIATED TOXICITY	SUGGESTED SUBSTITUTE
Abacavir (ABC)	Hypersensitivity reaction	AZT or TDF
Zidovudine (ZDV)	Severe anaemia or neutropenia<br>Severe gastrointestinal intolerance	TDF
	Lactic acidosis	TDF
Stavudine (d4T)	Lactic acidosis<br>Lipoatrophy/metabolic syndrome	TDF or ABC
	Peripheral neuropathy	AZT or TDF or ABC
Tenofovir (TDF)	Renal toxicity (renal tubular dysfunction)	AZT
Efavirenz (EFV)	Persistent and severe central nervous system toxicity	NVP or bPI or<br>ABC, if no other choice
	Potential teratogenicity (first trimester of pregnancy or women not using adequate contraception)	NVP or bPI or<br>ABC, if no other choice
Nevirapine (NVP)	Hepatitis	EFV or bPI or<br>ABC, if no other choice
	Hypersensitivity reaction	bPI or<br>ABC, if no other choice
	Severe or life-threatening rash<br>Stevens Johnson syndrome	Note: for non-life-threatening rash, EFV can be substituted

The general principle is that single-drug substitution because of toxicity should involve drugs belonging to the same ARV class. If toxicity is related to an identifiable drug in a regimen, the offending drug can be replaced with one that does not have the same side-effects (e.g. substitution of AZT or TDF for d4T in cases of neuropathy, TDF for AZT where anemia occurs, or NVP for EFV for CNS toxicity or in first trimester of pregnancy). Given the limited number of ARV drug options available in Nepal, drug substitutions should generally be limited to situations where toxicity is moderate to severe (grade 3) or life-threatening (grade 4). In a patient who experiences adverse effects, substitution of the offending drug is reasonable. In case of abacavir hypersensitivity reactions and nevirapine related hepatic failure or severe hypersensitivity, rechallenge should not be attempted as this may lead to toxicity and death.

Antiretroviral Treatment Failure and When to Switch Therapy

The decision on when to switch from first-line to second-line therapy is critical. If the decision is made too early the months or years of potential further survival benefit from many remaining first-line ARVs is lost; if it is made too late, the effectiveness of second-line therapy may be compromised and the patient is put at additional and appreciable risk of death. The time of switching is dictated by treatment failure, and this can be measured in three ways: clinically, by disease progression and WHO staging; immunologically, using trends in CD4 counts over time, and virologically, by measuring HIV viral loads (plasma HIV-1 RNA levels). Definitions of clinical and CD4-related treatment failure are given below. Nepal will continue to heavily rely on clinical and CD4 count criteria, in order to define treatment failure. It has been recognized that, treatment failure is recognized later solely on the basis of clinical and/or CD4 criteria, thus providing a greater opportunity for drug resistance mutations to evolve before regimen change. This can compromise the use of alternative regimen through drug class cross-resistance. Therefore, viral load will be used when possible to assist with diagnosis of treatment failure (see below). It is not likely that drug resistance testing will become a routine part of clinical care in Nepal in the foreseeable future and so is not considered in these recommendations. In all cases, adherence counseling is indicated and clinical judgment should be included in decision-making.

Treatment Failure

Three types of treatment failures (clinical, immunologic and virologic) have been identified.

Clinical Disease Progression as an Indicator of Failure:

Clinical disease progression with development of an opportunistic infection or malignancy when the drugs have been given sufficient time to induce a protective degree of immune restoration is a strong indicator of treatment failure. It should not be concluded, on the basis of clinical criteria, that an ARV regimen is failing until there has been a reasonable trial of first-line therapy lasting at least six to twelve months, adherence has been assessed and optimized, intercurrent opportunistic infections have been treated and resolved, and IRIS has been excluded. IRIS can be seen within the first several weeks after the institution of therapy, if a sub-clinical infection is present at baseline. It is also possible that this immunological reconstitution may lead to the development of atypical presentations of some opportunistic infections. In IRIS, changing the antiretroviral regimen is not indicated. The development of a new or recurrent WHO stage 3 or 4 condition on treatment (but after the first six months of ART) is considered functional evidence of HIV disease progression. TB can occur at any CD4 level and does not necessarily indicate ART failure. The response to TB therapy should be used to evaluate the need to switch ART. with pulmonary TB and some extrapulmonary TB diagnoses (e.g. simple lymph node TB or patients with uncomplicated pleural disease), where a good response to TB therapy is frequently seen, the decision to switch ART can be postponed and monitoring can be increased. This also applies if severe and/or recurrent bacterial infections or oesophageal candidiasis respond well to therapy.

Immunologic Failure:

The CD4 cell count remains the strongest predictor of HIV-related complications, even after the initiation of therapy. The baseline pretreatment value is informative: lower CD4 counts are associated with smaller and slower improvements in count. Patients starting with low CD4 counts may demonstrate slow recovery, but persistent levels below 100 cells/mm^3 represent significant risk for HIV disease progression. It should be noted that intercurrent infections can result in transient CD4 count decreases. As a general principle, intercurrent infections should be managed, time should be allowed for recovery and the CD4 cell count should be measured before ART is switched. If resources permit, a second CD4 cell count should be obtained to confirm immunological failure.

Definitions of immunological failure are: (1) CD4 count below 100 cells/mm^3 after six months of therapy; (2) a return to, or a fall below, the pre-therapy CD4 baseline after six months of therapy; or (3) a 50% decline from the on-treatment peak CD4 value. The CD4 cell count can also be used to determine when not to switch therapy, e.g. in a patient with a new clinical stage 3 event for whom switching is being considered or in a patient who is asymptomatic and under routine follow-up.

Plasma Viral Load as an Indicator of Treatment Failure:

Viral load testing, when available is a sensitive and informative way to identify treatment failure. When treatment failure is defined on the basis of clinical and/or CD4 criteria the diagnosis may be made later than when viral load is being monitored. The viral load threshold triggering a switch in ART is defined as persistent viral load >5,000 after 6 months on ART. An undetectable viral load mandates that ART should not, in general, be switched irrespective of the CD4 cell count or the clinical stage.

All clients being considered for second-line therapy will have their case reviewed by certified National HIV experts. Both decisions of when to change therapy and what regimen to change to should be made in consultation with experts.

Table 10: Clinical, CD4 Cell Count and Virological Definitions of Treatment Failure

**CLINICAL FAILURE**[a]	New or recurrent WHO stage 4 condition[b]
**CD4 CELL FAILURE**[c]	Fall of CD4 count to pre-therapy baseline (or below);
	Or 50% fall from the on-treatment peak value (if known);
	Or persistent CD4 levels below 100 cells/mm^3
**VIROLOGICAL FAILURE**	Persistent plasma viral load above 5,000 copies/ml

[a]Current event must be differentiated from the immune reconstitution inflammatory syndrome.
[b]Certain WHO clinical stage 3 conditions (e.g. pulmonary TB, severe bacterial infections), may be an indication of treatment failure.
[c] without concomitant infection to cause transient CD4 cell decrease.

Clinical status, the CD4 cell count, and the plasma HIV-1 RNA level (if available) can be used in an integrated fashion to determine whether HIV disease is progressing on therapy and whether a change from first-line to second-line therapy should be made. See table below for guidance. Clinical judgment remains an important part of the decision-making process.

Changing Antiretroviral Treatment for Failure

In case of treatment failure, the entire regimen should be changed from a first to a second line combination regimen. A single drug should not be added or changed to a failing regimen. The new second-line regimen will need to use drugs which retain activity against the patient's virus strain and a minimum of three active drugs, one of them drawn from at least one new class, in order to increase the likelihood of treatment success and minimize the risk of cross-resistance. The PI class is thus reserved for second-line treatment. Ritonavir-boosted protease inhibitors are preferred. They should be supported by new agents from the NRTI class. Patients should not switch from one NNRTI to the other at the time of failure, as there is a high chance of cross-resistance (ie. do not give EFV after NVP or vice versa).

Recommended Second-line Regimens in Adults and Adolescents

For Failure on:		Change to:
ZDV or d4T + 3TC + NVP or EFV	→	TDF + 3TC + LPV/r[a]
TDF + 3TC + NVP or EFV	→	ZDF + 3TC + LPV/r[a]

[a]Adult LPV/r tablets do not require cold chain. If a heat-stable affordable version of Atazanavir/ ritonavir (ATV/r) becomes available, this will be a good choice for use in Nepal. Alternative PI options include ATV/r, but the current formulation requires refrigeration.

When ZDV or d4T was used in the first line, the best possible second-line regimen is TDF/3TC. The issues of drug hypersensitivity with ABC remain as does the fact that high-level ZDV/3TC co-resistance confers diminished susceptibility to ABC. TDF often retains activity against nucleoside-resistant viral strains. It is attractive because it is administered once daily. TDF should not be used in combination with ddI due to decreased virologic efficacy and increased toxicity. Recycling 3TC is now recommended to prolong the common 3TC mutation which makes the virus "less fit" as one of the best options for second-line therapy.

Because of the diminished potential of almost any second-line nucleoside component, a ritonavir-boosted PI (bPI) component, i.e. lopinavir (LPV/r) or atazanavir (ATV/r), is preferred. Of these, currently, only LPV/r is available in a heat-stable form, which does not require refrigeration, although ATV/r heat stable version is under development.

For treatment failure with a first-line PI-based regimen, the choice of an alternative regimen depends on the reason for the initial choice of a PI-based, rather than an NNRTI-based, regimen. If the reason was suspected NNRTI resistance or HIV-2 infection the choice of the alternative regimen is not straight forward. In these situations the options depend on the constraints imposed by the circumstances of individual patients, the capabilities of individual managements to test for resistance to drugs, and the ARV formulary in Nepal.

Treatment failure on a triple NRTI regimen is more easily managed because two important drug classes (NNRTIs and PIs) will have been spared. Thus a boosted PI + NNRTI +/- alternative NRTIs (e.g. ddI and/or TDF) can be considered if drug availability permits.

Patients on a failing second-line regimen with no new ARV options should continue on a tolerated regimen.

Note: Didanosine and Abacavir are no longer part of recommended second-line regimens due to increased cost and complexity. In addition, ABC cross resistance is common after ZDV or d4T use in first-line. However ddI and ABC should continue to be available in Nepal for the use in triple NRTI combinations and other circumstances where standard NRTIs are contraindicated.

VIII. Monitoring of HIV Drug Resistance

Individual HIV drug resistance (HIVDR) testing to guide treatment is not recommended.

Monitoring should be Done Using:

- "Early Warning Indicators" for HIV drug resistance targeted at ART facilities, which are designed to alert programme managers to programmatic factors that are likely to be associated with poor outcomes of ART.
- Monitoring surveys to assess the emergence of HIVDR and associated factors in cohort (s) of treated patients 12 months after ART initiation in sentinel ART sites.
- Surveys of representative samples of subjects for the presence of transmitted HIVDR
- Surveys of representative samples of subjects for the presence of acquired HIVDR

For details on the Early Warning Indicators for the country, see Annex VII.

IX. Discontinuation or Interruption of Antiretroviral Therapy

Discontinuation of ART may result in viral rebound, immune decompensation, and clinical progression. **Planned ART interruptions are NOT recommended.** Unplanned interruption of ART may become necessary because of severe drug toxicity, intervening illness, surgery that precludes oral therapy, or antiretroviral medication nonavailability. More commonly patients return for care after having stopped their medication on their own, against medical advice for a variety of reasons.

PMTCT recommendations now include triple ARVs for pregnant or breastfeeding mothers who do not yet need life-long ART for their own health. These should be discontinued 1 week after complete cessation of breastfeeding. If the regimen includes an NNRTI such as EFV, the dual NRTI backbone should be continued for 1 week beyond the discontinuation of EFV. See most recent national PMTCT guidelines.

What to Do in Different Scenarios:

- Stopping antiretroviral drugs for a short time (i.e. <1 to 2 days) due to medical/surgical procedures can usually be done by holding all drugs in the regimen.
- When a patient experiences a severe or life-threatening toxicity or unexpected inability to take oral medications- all components of the drug regimen should be stopped simultaneously, regardless of drug half-life.
- All discontinued regimen components should be restarted simultaneously
- **Discontinuation of Efavirenz or Nevirapine**. For combinations of NRTIs + NNRTI, the longer half-life of the NNRTI may lead to functional monotherapy if all drugs are discontinued together. This may increase the chance of NNRTI-resistant mutations. The NRTI back-bone can be continued for at least one week after NVP or EFV is stopped to reduce this. An alternative strategy is to substitute a PI for the NNRTI and to continue the PI with dual NRTIs for a period of time.

- **Reintroduction of Nevirapine Using Dose Escalation**. Because nevirapine is an inducer of the drug-metabolizing hepatic enzymes, administration of full therapeutic doses of nevirapine without a 2-week, low-dose escalation phase will result in excess plasma drug levels and potentially increase the risk for toxicity. Therefore, in a patient who has interrupted treatment with nevirapine for more than 2 weeks, nevirapine should be reintroduced with a dose escalation period of 200mg once daily for 14 days, then a 200mg twice-daily regimen.
- **Discontinuation of Lamivudine or Tenofovir in Patients with Hepatitis B Coinfection**. Patients with hepatitis B coinfection who are receiving one or both of these NRTIs may experience an exacerbation of hepatitis upon drug discontinuation

X. Antiretroviral Therapy in Pregnancy

ARV regimens given for treatment to pregnant women should preferably include drugs shown to be effective in reducing mother-to-child transmission. First-line treatment regimens in pregnant women should include ZDV whenever possible. The combination of ZDV/3TC has been recommended as the first choice for use in pregnancy. If hemoglobin monitoring is not possible or baseline hemoglobin is low, use TDF in place of ZDV in pregnancy. There is potential increased risk of lactic acidosis with the combination of d4T/ddI in pregnant women, so this combination should be avoided. NVP is the choice NNRTI for use in pregnancy, but should not be prescribed with CD4 over 250. EFV is contraindicated in the first trimester of pregnancy. PIs have been associated with the development of glucose intolerance and even diabetes mellitus in non-pregnant individuals. Among PIs, Lopinavir/ritonavir is the first choice PI for use during pregnancy. Antiretroviral therapy should be started as soon as possible in pregnancy. ART should not be stopped during first trimester due to risk of viral rebound and decline in CD4, risking maternal disease progression and increasing transmission.

Prevention of Mother-to-Child Transmission (MTCT) of HIV

The area for PMTCT of HIV is one of the most rapidly evolving in all of HIV care. Please refer to the most up-to-date version of Nepal National Guidelines.

Comprehensive 4 Prong Strategy for PMTCT of HIV

1. Prevent HIV Infection among Women of Child-bearing age.
2. Prevent unintended pregnancies among women living with HIV
3. Prevent HIV transmission from HIV infected mothers to their infants
    1. Antiretroviral prophylaxis for mother and baby
    2. Safer delivery practices
    3. Safer infant feeding
4. Provide appropriate treatment, care and support to women living with HIV and their children and families

When to Commence Life Long ART in Pregnancy: as in other non-pregnant adults

Initiate highly active antiretroviral therapy (life-long ART) in any pregnant woman with:

- WHO Stage 4 disease, irrespective of CD4 cell count
- WHO Stage 3 disease, irrespective of CD4 cell count
- WHO Stage 1 or 2 disease with CD4 count less than 350/mm^3

Note: ART is even more urgent in the case of pregnant women, given the potential to decrease HIV transmission to the infant and potential NVP resistance issues from short course ARVs. Pregnant women needing ART should start ART as soon as possible.

The Standard Life-long ART Regimen in Pregnancy is: ZDV + 3TC + NVP

This should be used in those women eligible by the above criteria. Efavirenz can be used after the first trimester of pregnancy and may be preferred in women with higher CD4 counts. NVP should not be used in those with CD4 over 350 and only used with caution in those between 250 and 350 due to risk of liver toxicity. All pregnant women taking NVP need close monitoring of liver function. If close liver function monitoring is not possible, consider using EFV instead of NVP after the first trimester, for those with CD4 over 250.

Table 11: Recommended First-Line Life-long ART Regimen for Treating Pregnant Women, and Prophylactic Regimen for Infants

Recipient	Timing	ARV(s)		
**Mother**	Start ASAP and continue throughout pregnancy, labour and delivery and postpartum, for life	**ZDV 300mg bid + 3TC 150mg bid + NVP 200mg od**<br>If CD4 <250 or 250-350 with liver monitoring available<br>If no reaction, continue ZDV + 3TC and increase NVP to 200mg twice a day after 14 days		
		**ZDV 300mg bid + 3TC 150 mg bid + EFV 600mg od**<br>If CD4 is between 250-350 with no liver monitoring available or CD4 >350		
**Baby**	Neonatal	**Infant NVP syrup (10mg/ml) given daily from birth to 6 weeks**		
		**Birth Weight**	**Dose**	**Quantity**
		<2.5kg	10mg/d	1ml
		>2.5kg	15mg/d	1.5ml

Note: Due to increased chance of NVP induced hepatotoxicity, EFV is preferred after the first trimester in women with:

- CD4 between 250 and 350 unless LFTs can be monitored closely.
- CD4 over 350 (regardless of lab capabilities)

Other options for the third ARV in this group include Abacavir or Lopinavir/ritonavir

Substitute TDF for ZDV if anemic or unable to monitor haemoglobin.

Antiretroviral Prophylaxis for Mother and Baby (When Mother does Not Yet Need ART)

The risk of HIV transmission to the baby can be reduced to 5% or less, if the mother takes ARVs during the antenatal period and with careful management of the delivery and provision of ARVs to both mother and baby after delivery and to mother throughout breastfeeding.

Recommended PMTCT Prophylactic Short-term Triple Antiretrovirals

All HIV positive pregnant or lactating women should be seen at the ART clinic. All those not yet eligible for life-long ART should be offered short term triple antiretroviral prophylaxis starting from as early as 14 weeks of pregnancy (or when first seen, if later in pregnancy). The mother should take triple antiretrovirals during the second and third trimester of pregnancy (from 14 weeks) and for one year postpartum while breastfeeding her baby. Antiretrovirals should be stopped one week after breastfeeding cessation, usually at 12 months of age.

Table 12: Preferred Regimen of Triple ARV Prophylaxis for Mother and Baby

<table>
<tr><th>Recipient</th><th>Timing</th><th colspan="3">ARV(s)</th></tr>
<tr><td rowspan="1">Mother</td><td>■ Antenatally from 14 weeks
■ During labour
■ Postpartum for 1 year until 1 week after breastfeeding cessation</td><td colspan="3">ZDV 300mg bid + 3TC 150 mg bid + EFV 600mg od

For anaemic women or where Hb monitoring is not possible, use TDF in place of ZDV.
ABC or LPV/r can be substituted for EFV if needed.
Note for mothers on EFV: At discontinuation of triple ARVs, woman should stop EFV first. She should continue taking only 2 NRTI backbone (usually ZDV+3TC) for 1 week to prevent NNRTI resistance.</td></tr>
<tr><td rowspan="4">Baby</td><td rowspan="4">Neonatal</td><td colspan="3">Infant NVP syrup (10mg/ml) given daily from birth to 6 weeks</td></tr>
<tr><td>Birth Weight</td><td>Dose</td><td>Quantity</td></tr>
<tr><td><2.5kg</td><td>10mg/d</td><td>1ml</td></tr>
<tr><td>>2.5kg</td><td>15mg/d</td><td>1.5ml</td></tr>
</table>

Infant Issues Including Infant feeding:

Nevirapine

All HIV-exposed infants should receive daily nevirapine from as soon as possible after birth to 6 weeks of life (regardless of what the mother received).

Breast-feeding

Breastfeeding, in particular exclusive breastfeeding, is the ideal way to feed infants and it should be protected, promoted and supported. Beyond sound nutrition, it protects against common childhood infections. However, it is one of the routes for mother-to-child HIV transmission. Nepal experts agree with WHO in the promotion of breastfeeding along with the use of antiretroviral prophylaxis as the ideal way to feed infants of HIV-infected women in Nepal.

Breastfeeding is Now Recommended for HIV-exposed Babies Until 12 Months of Age

Mothers should exclusively breastfeed their infants for the first 6 months of life, introducing appropriate complementary foods thereafter, and continue breastfeeding for the first 12 months of life while taking triple ARV prophylaxis throughout.

12 months of breastfeeding gives the maximum survival benefit of breastfeeding. Mothers take ARVs to reduce the risk of transmission during the entire duration of breastfeeding. After 12 months of age, the survival benefits of breastfeeding decreases. Breastfeeding should then only stop once a nutritionally adequate and safe diet without breast milk can be provided.

Breastfeeding Cessation

HIV-infected mothers should stop breastfeeding gradually within one month. Mothers should continue triple ARV prophylaxis for one week after breastfeeding is fully stopped. Stopping breastfeeding abruptly is not advisable.

Research shows that rapid and abrupt cessation breastfeeding is associated with adverse consequences for the infant such as growth failure and increased prevalence of diarrhoea.

Breast milk viral load is also known to spike with rapid cessation of breastfeeding, so there is chance of increased transmission if the child is fed again.

ARV Prophylaxis During Breastfeeding

Mothers should continue triple ARV drugs until one week after all exposure to breast milk has ended.

If a mother is taking an NNRTI (most commonly EFV), the NNRTI should be stopped one week after breastfeeding cessation and the two NRTI ARVs continued for an additional one week. This is to decrease the chance of NNRTI resistance, which is more likely when all three drugs are stopped together.

All women with HIV should be offered contraception at 6 weeks postpartum. If adequate contraception can not be assured, EFV should be changed to either ABC or LPV/r while the NRTI backbone is continued. EFV is teratogenic in the first trimester of pregnancy and must be avoided in any woman with a chance of another pregnancy.

If a breastfeeding mother stops triple ARVs for whatever reason, provider should consider giving the baby daily NVP until one week after breastfeeding cessation according to the following dosing table.

Table 13: Prophylactic Nevirapine for Breastfed HIV-exposed Infants

NVP Infant Dosing Guide NVP syrup (10mg/ml)

Age	Dose	Quantity
Birth to 6 weeks <2.5kg birth weight	10mg/d	1ml
Birth to 6 weeks >2.5kg birth weight	15mg/d	1.5ml
6 weeks to 6 months	20mg/d	2ml
6 months to 9 months	30mg/d	3ml
9 months to end BF	40mg/d	4ml

Anaemia in Pregnancy

ZDV may cause anaemia and neutropenia. Investigate for and treat any underlying causes of severe anaemia (Hb <7 g/dl) or neutropenia. Other NRTI drugs (TDF) may be substituted for ZDV.

Prong 2 of PMTCT: HIV/ART and Family Planning Options

To avoid unintended, unplanned pregnancies among HIV positive couples, careful reproductive health and family planning counseling is essential for all people living with HIV.

Condoms: Male (or female) condoms are the only contraceptive method that can provide "dual protection" against STIs and HIV, and are therefore always recommended where one or both partners are infected with HIV.

Table 14: Medical Eligibility for Contraceptive Methods for Clients with HIV and AIDS

Contraceptive Method		Condition				
		HIV-infected	AIDS	ARV Therapy		
				NRTIs	NNRTIs	Ritonavir
DMPA		1	1	1	1	1
NET-EN		1	1	1	2	2
Implants		1	1	1	2	2
Oral Contraceptives		1	1	1	2	3
IUCD	Starting	2	3*	2/3*	2/3*	2/3*
	Ongoing	2	2	2	2	2
Condoms		No restrictions: use with hormonal contraception is encouraged to prevent HIV/STI transmission				
ECPs		No restrictions				
Sterilisation		No reasons to deny; delay in cases of acute HIV infection				
FAB Methods		Can use if menstrual cycle is regular. Encourage continued use of condoms outside the fertile window to prevent STI/HIV Transmission				
LAM		Advise on the risk of transmission; exclusive breastfeeding reduces rise compared to mixed feeding				
Spermicides and Diaphgram		Use is not recommended may increase risk of HIV transmission/superinfection.				
**Definitions**						
Catagory 1: No restrictions on use						
Catagory 2: Benefits generally outweight the risks						
Catagory 3: Risks generally outweight the benefits: seek specialist advice before using						
* Category 2 if client with AIDS is clinically well on ARV therapy; otherwise category 3.						

Note: DMPA (Depo Provera) and NET-EN are Injectable progesterone methods. In 2012 WHO added a clarification that women at risk for HIV can use these methods, but should be strongly encouraged to also use condoms.

ECP: Emergency Contraceptive Pills, FAB: Fertility Awareness Based method, LAM: Lactational Ammenorhea Method

Infant Feeding Recommendations

Recommendations on Infant Feeding to Babies Born to HIV Positive Mothers:

- Breast feeding is recommended for all babies of HIV-infected mothers for 12 months.
- Exclusive breast feeding is recommended for all infants of HIV-infected woman for the first six months.
- After 6 months, culturally appropriate complimentary foods (weaning) is started and mother is advised to stop breast feeding as early as possible.
- Babies should continue to breastfeed while adding complementary feeds from 6 to 12 months.
- Babies of HIV-infected mothers should stop breastfeeding at about 12 months of life, if it is safe to do so.
- Conditions for safe replacement feeding should be assessed prior to 12 month postpartum weaning.
- Breastfeeding should be stopped gradually over 1 month
- ARV prophylaxis should be continued until 1 week after all breastfeeding stops. Women who do not yet require ART for their own health can stop triple ARV prophylaxis then.
- If a child is diagnosed as HIV-infected, the mother should be encouraged to continue breastfeeding the child until at least 2 years of age.

XI. Tuberculosis and Antiretroviral Therapy

The life time risk of someone with latent TB developing TB disease in an HIV negative individual is 5-10% whereas PLHIV have up to a 50% risk. Therefore managing TB among HIV infected individuals is one of the major responsibilities of the ART clinician.

Intensified Case Finding

As TB is one of the most common opportunistic infection among the HIV infected people, all clients diagnosed HIV positive in VCT centers and all HIV positive people visiting ART centers should be screened for TB using a standard screening process.

Screening Questions for Active TB:

Does patient currently have any of the following:

1.	Cough?	Yes	No
2.	Fever?	Yes	No
3.	Weight loss?	Yes	No
4.	Night sweats?	Yes	No

If "Yes" to any of the 4 questions, further TB investigation is needed.

Ask the client to report immediately if any of the above mentioned symptoms occur.

Isoniazid Preventive Therapy (IPT)

IPT refers to taking 6 months of isoniazid daily for latent TB infection regardless of CD4 cell count or ART status.

Identifying Those in Need of IPT:

- Perform TB screening for all new HIV infected clients at their first visit with 4 TB screening questions, a full initial history and physical examination. Repeat screening should take place at 3 monthly intervals for all PLHIV.
- Anyone answering "yes" to any of the four screening questions should be sent for investigations as needed.
- If there are any signs of active TB or any concerns about unexplained illness, do NOT offer IPT, but refer client to TB doctor or supervising doctor as appropriate.
- All PLHIV without active TB or other unexplained illness are offered IPT with appropriate counseling.

Initiating IPT:

- Explain the IPT program to the client and assess predicted adherence to 6 months of Isoniazid.
- Cotrimoxazole and ART should not be started at the same time as IPT.
- Those with liver disease, active alcohol use, jaundice, habitual treatment defaulter and prior Isoniazid resistance should be excluded.
- DOTS is not needed for IPT

IPT Regimen:

Isoniazid 300 mg daily for 6 months. If available, Vitamin B6 25 mg (pyridoxine) should be given together with IPT for 6 months.

Follow-up Visits While on IPT:

- Client must be seen every month for adherence check, side effect check and medication refill.
- Ask about symptoms of breakthrough TB at each visit. If any occur, evaluate for TB.

See latest Nepal IPT guidelines for more details.

Patterns of HIV-related TB

Pulmonary TB (PTB)

Even in HIV-infected patients, PTB is still the commonest form of TB. The presentation depends on the degree of immunosuppression. The table below shows how the clinical picture, sputum smear result and CXR appearance often differ in early and late HIV infection.

Table 15: How PTB Differs in Early and Late HIV Infection

Features of PTB	Stage of HIV Infection	
	Early	Late
**Clinical Picture**	Often resembles post-primary PTB	Often resembles primary PTB
**Sputum smear result**	Often positive	Often negative
**CXR appearance**	Often cavities	Often infiltrates with no cavities

Extrapulmonary TB

The most common forms of extrapulmonary TB are: pleural effusion, lymphadenopathy, pericardial disease, miliary disease, meningitis, disseminated TB (with mycobacteraemia).

TB Treatment in HIV

Effective treatment and control of TB is a central priority when developing treatment strategies for co-infected patients. Tuberculosis treatment following the DOTS strategy should be initiated promptly in diagnosed cases of TB. All PLHIV with TB need ART. Suggestions for timing of initiation of ART for TB patients in different situations is given in following table:

Table 16: Strategy for Initiation of Treatment for Both TB and HIV Infection

1. Start ART in all PLHIV with active TB, irrespective of CD4 count.
2. Start TB treatment first, followed by ART as soon as possible thereafter, but by 8 weeks at the latest.
3. Use Efavirenz as the preferred NNRTI in TB-HIV coinfection

ARV Drug Choice in TB Co-infection:

- First line treatment option is ZDV/3TC or TDF/3TC plus Efavirenz (600mg once daily).
- Dose increase of Efavirenz is no longer recommended during ATT.
- Alternative regimens include either:
    - ZDV/3TC or TDF/3TC plus NVP (start bid NVP, no dose escalation) OR
    - ZDV/3TC or TDF/3TC plus Abacavir.

Note that Rifampicin decreases Nevirapine levels by hepatic induction. There are also concerns of additive liver toxicities. However, with close monitoring NVP containing regimens may be considered. One exception is that women with baseline CD4 >250 should not be given NVP along with Rifampicin. NVP should be started at 200mg bid (no lead in dose) if patient is taking Rifampicin.

Patients already receiving ART when they develop TB should adjust the regimen to be compatible with TB treatment. Following completion of anti-tubercular therapy the ART regimen can be continued or changed depending upon the clinical and immunologic status of the patient.

Second line ART and TB-coinfection: Drug choices: Use of Rifabutin in TB-HIV co-therapy.

The HIV pandemic has led to a resurgence of tuberculosis and the challenge of TB-HIV co-therapy for patients on second-line ART is well recognized. Management of co-infected patients has shown that TB can be cured with standard antituberculosis regimens, including the use of rifampicin-based TB treatment for 6 months. Preliminary evidence and experience has confirmed recommendations in WHO guidelines that for most patients, especially those with CD4 counts < 100 cells/mm^3, HIV treatment should not be delayed, but should be started or continued along side TB treatment. It is expected that many patients will fail first-line ART with active TB; and TB will develop in patients on second-line therapy. However, because of well recognized drug-drug interactions, it is difficult to use Rifampicin with any boosted PI-based regimens. For patients who need antituberculosis treatment in Nepal and who need a boosted PI, the only option is:

Substitute rifabutin for rifampicin in the anti-TB regimen and maintain the standard PI-based ART regimen. Dose of rifabutin when used with boosted PI is 150mg three times a week.

XII. Hepatitis C Co-infection and ART

Hepatitis C patients should be monitored by following ALT regularly. If ALT is elevated, check full liver function tests. If transaminases are >5 times the upper limit of normal, carefully evaluate for signs and symptoms of liver insufficiency and for alternative causes of liver injury (e.g., acute viral hepatitis A or B infection, hepatobiliary disease, or alcoholic hepatitis); short-term ART interruption may be required.

Rarely, PLHIV may access Hepatitis C treatment in Nepal. Concurrent treatment of both HIV and HCV is feasible but may be complicated by pill burden, drug toxicities, and drug interactions such as the following:

- Didanosine should not be given with ribavirin because of the potential for drug-drug interactions leading to life-threatening ddI-associated mitochondrial toxicity including hepatomegaly/steatosis, pancreatitis, and lactic acidosis
- Zidovudine should be avoided with ribavirin when possible because of possible anemia. If no alternatives, ribavirin dose reduction is necessary.
- Drug-induced liver injury from ART is more common in HIV /HCV coinfection.
- Stavudine (with or without didanosine) and nevirapine are the ARVs most likely to cause liver injury.

XIII. Hepatitis B Co-infection and ART

All PLHIV with chronic active Hepatitis B co-infection should be started on ART irrespective of CD4 cell count or WHO clinical stage. All should be started on TDF + 3TC as the NRTI backbone. At least 2 ARVs which are active against Hepatitis B should be given.

First line ART for those with Hep B coinfection: TDF+3TC+EFV

Second line ART for those with Hep B coinfection: AZT+TDF+3TC+LPV/r (maintain TDF+3TC and add extra NRTI plus PI)

Beware of potential for liver flare if 3TC or TDF are discontinued.

XIV. Immune Reconstitution Inflammatory Syndrome (IRIS)

Immune Reconstitution Inflammatory Syndrome is a spectrum of clinical signs and symptoms resulting from the restored ability to mount an inflammatory response associated with immune recovery. It can present with the signs and symptoms of a previously subclinical and unrecognized opportunistic infection, as a paradoxical worsening of treatment response several weeks into therapy, or as an autoimmune disease in the context of immune recovery on ART. Typically, IRIS occurs within two to twelve weeks of the initiation of ART, although it may present up to 24 weeks after ART initiation. The incidence of IRIS is estimated to be 10%-32% of adults initiating ART. There is a higher risk in those starting ART with lower CD4 counts.

The syndrome can be characterized by fever, lymphadenopathy, worsening pulmonary lesions (on X-ray examination), expanding central nervous system (CNS) lesions, elevation of hepatic enzymes (Hep B co-infection), skin lesions or signs of autoimmune diseases. Sometimes, a brief course of corticosteroids may be required to reduce inflammation for severe respiratory or CNS symptoms. Prednisolone (or prednisone) at 0.5 mg/kg/day for five to ten days is suggested in moderate to severe cases of IRIS. Steroid doses have to be adjusted upwards when using together with microsomal enzyme enhancers like Rifampicin.

XV. Cotrimoxazole Prophylaxis in Adults

All PLHIV should be evaluated for possible need for Cotrimoxazole prophylaxis, even in areas without ART accessibility, or in the time that they are preparing for ART initiation. Cotrimoxazole has been proven to prevent morbidity and mortality in PLHIV and is a very important component of a complete package of care for HIV.

Cotrimoxazole Prophylaxis should be Given to:

- HIV infected adults with CD4 count <350 cells/mm^3
- All adults who have had an episode of PCP
- All adults with symptomatic HIV disease or Clinical stage 2, 3 or 4

The Regimen is:

1 DS tablet (160TMP/800SMX) every day OR

2 SS tablets (80TMP/400SMX) every day

1. Continue Cotrimoxazole Prophylaxis as Follows:

- Lifelong, if not on ART
- If on ART the CD4 is >350 on two consecutive samples 6 months apart, Cotrimoxazole can be discontinued.
- Stop prophylaxis for severe cutaneous reactions, such as Stevens-Johnson syndrome, renal and/or hepatic failure, and severe hematological toxicity.

2. Timing of Cotrimoxazole Prophylaxis in Relation to ART Initiation: Since the most common initial side effect of cotrimoxazole and antiretroviral therapy (especially nevirapine and efavirenz) is rash, it is recommended to start cotrimoxazole prophylaxis first and to initiate antiretroviral therapy two weeks later if the individual is stable on cotrimoxazole and has no rash. Do NOT start Cotrimoxazole and ART at the same time.

3. For Cotrimoxazole Intolerance, Consider The Following Alternatives:

- Dapsone 100 mg once daily is the first choice.

  OR

- In cases of non-life-threatening adverse reactions, stop treatment for two weeks; then re-challenge the client with cotrimoxazole in a gradually increasing dose of an oral suspension of cotrimoxazole. After desensitization under surveillance, up to 70 percent of clients may again tolerate cotrimoxazole.

Table 17: Protocol for Cotrimoxazole Desensitization among Adults and Adolescents

STEP	DOSE
DAY 1	80mg sulfamethoxazole + 16mg trimethoprim (2 ml of oral suspension[a])
DAY 2	160mg sulfamethoxazole + 32mg trimethoprim (4 ml of oral suspension[a])
DAY 3	240mg sulfamethoxazole + 48mg trimethoprim (6 ml of oral suspension[a])
DAY 4	320mg sulfamethoxazole + 64mg trimethoprim (8 ml of oral suspension[a])
DAY 5	One single-strength sulfamethoxazole-trimethoprim tablet (400mg sulfamethoxazole + 80mg trimethoprim)
DAY 6 ONWARDS	Two single-strength sulfamethoxazole-trimethoprim tablets or one double strength tablet (800mg sulfamethoxazole + 160 mg trimethoprim)

[a]Cotrimoxazole oral suspension is 40mg trimethoprim + 200mg sulfamethoxazole per 5 ml.

1. Follow-up of clients on Cotrimoxazole prophylaxis every month.

- Monitor for toxicity, clinical events and adherence.
- Lab tests of hemoglobin and white blood counts, only as indicated.

2. Adherence counseling on Cotrimoxazole can be useful to help prepare clients for ART in the future and problem-solve barriers to medication adherence.

3. If prophylaxis has been stopped because of immune improvement, Cotrimoxazole prophylaxis (or Dapsone) should be recommenced if the CD4 cell count falls below 350 or if new or recurrent WHO clinical stage 2, 3 or 4 conditions occur.

4. Use an alternative antibiotic for treating breakthrough bacterial infections among individuals living with HIV receiving cotrimoxazole prophylaxis, while continuing cotrimoxazole.

5. For toxoplasmosis and PCP infections, prophylaxis should be suspended and full active treatment initiated. Cotrimoxazole prophylaxis should be recommenced after the treatment course.

XVI. Post Exposure Prophylaxis (PEP) of HIV

Post Exposure Prophylaxis (PEP) is currently the only way to reduce the risk of HIV infection in someone exposed to the virus. It refers to the use of antiretroviral medications to help prevent HIV transmission. The rationale is that ARVs given immediately after exposure can stop the virus from disseminating in the body and establishing infection.

The majority of occupational exposures do not lead to HIV infection. The risk of HIV transmission following skin puncture from a needle or other sharp object that was contaminated with a blood from a person with "documented" HIV infections is about 0.3%. The risk of HIV transmission is less with injuries sustained with solid bore (e.g. suture) needles than with hollow bore (e.g. blood drawing) needles. Similarly, the smaller the size of hollow bore needle, the less risk of HIV transmission. There have been rare reports of health workers who have become infected by exposure of mucous membrane (of eyes, nose or mouth) or abraded (broken) skin to HIV-infected material; the risk is estimated to about 0.09%. HIV is not transmitted through healthy intact skin.

1. First Aid Immediately after Potential Exposure:

The aim of first aid is to reduce contact time with the source person's blood, body fluids or tissues and to clean and decontaminate the site of the exposure.

If the skin is broken following an injury with a used needle or sharp instrument, the following is recommended.

- Do not squeeze or rub the injury site.
- Wash the site immediately using soap or a mild disinfectant solution that will not irritate the skin.
- If running water is not available, clean the site with a gel or other hand-cleaning solution, whatever is customarily available.
- Do not use strong solutions, such as bleach or iodine, to clean the site as these may irritate the wound and make the injury worse.

After a splash of blood or body fluids on broken skin, the following is recommended:

- Wash the area immediately.
- If running water is not available, clean the area with a gel or other hand-rub solution, whatever is customarily available.
- Do not use strong disinfectants.

After a splash contacts the eye, do the following:

- Irrigate the exposed eye immediately with water or normal saline.
- Sit in a chair, tilt the head back and have a colleague gently pour water or normal saline over the eye, pulling the eyelids up and down to make sure the eye is cleaned thoroughly.
- If contact lenses are worn, leave these in place while irrigating the eye, as they form a barrier over the eye and will help protect it. Once the eye has been cleaned, remove the contact lenses and clean them in the normal manner. This will make them safe to wear again.
- Do not use soap or disinfectant on the eye.

After a splash contacts the mouth, do the following.

- Spit the fluid out immediately.
- Rinse the mouth thoroughly, using water or saline, and spit again. Repeat this process several times.
- Do not use soap or disinfectant in the mouth

Indications for Post Exposure Prophylaxis

1. The exposed person is HIV-negative
2. The source person is HIV positive, or at high risk of recent infection and thus likely to be in the window period.
3. The exposure poses a risk of transmission, that is:
    a. Percutaneous exposure to potentially infectious body fluids
        i. (non-infectious body fluids include faeces, saliva, urine and sweat)
    b. Sexual intercourse without an intact condom
    c. Exposure to non-intact skin or mucus membranes to potentially infectious body fluids
4. The exposure occurred less than 72 hours previously.
5. The exposure is not part of chronic exposure (prevention support needed instead)

Whether to Give PEP and Which Type of Regimen:

The decision to start PEP is made on the basis of degree of exposure to HIV and HIV status of the source from whom exposure has occurred (source patient). Decisions should be made based in part on information about the source, including ART, response to therapy, including viral load, and any data on HIV resistance testing. Decisions should not delay initiation of PEP, and modifications can be made after information is obtained. If the HIV status of the source is not known, HIV testing of the source could be done after necessary counseling. In any case, if the risk is high, PEP should be started immediately. If the HIV test results of the source are found to be negative, PEP can be discontinued. Specific recommendations for PEP are given in the two tables below, one for percutaneous injuries and the second for exposures to mucous membranes or non-intact skin.

Table 18: HIV PEP for Percutaneous Injuries

**Exposure**	**Status of Source**		
	**Low Risk***	**High Risk***	**Unknown**
**Not Severe:** Solid needle, superficial	2 drug PEP*	3 drug PEP*	Usually none: Consider 2 drug PEP
**Severe:** Large bore, deep injury, visible blood on device, needle in patient's artery/vein	3 drug PEP*	3 drug PEP*	Usually none: Consider 3 drug PEP

*Low risk: Asymptomatic HIV or VL <1500 c/ml. High risk: Symptomatic HIV/AIDS, acute seroconversion, high VL.

Concern for drug resistance: Initiate prophylaxis without delay and consult an expert.

Consider 3 drug PEP if source is high risk for HIV or exposure from unknown source when HIV infected source is likely.

Table 19: HIV PEP for Mucous Membrane and Non-intact Skin Exposures*

Exposure	Status of Sources		
	**Low Risk***	**High Risk***	**Unknown**
Small volume (drops)	Consider 2 drug PEP	2 drug PEP	Usually no PEP: Consider 2 drug PEP
Large volume (major blood splash)	2 drug PEP	3 drug PEP	Usually no PEP Consider 2 drug PEP

*Non-intact skin = Dermatitis, abrasion, wound

Low risk = Asymptomatic or VL <1500 c/ml High, risk = symptomatic HIV, AIDS acute seroconversion, high HIV viral load.

Consider 3 drug PEP, if source has HIV risk factors or exposure from unknown source where HIV infected source is likely.

Drug Selection for PEP

There are two types of regimens recommended for PEP. They are the basic regimen of two drugs combination and expanded regimen of three drugs as given below:

Regimens for PEP

2 DRUG COMBINATIONS (BASIC REGIMEN)

ZDV + 3TC

3 DRUG COMBINATIONS (EXPANDED REGIMEN)

2 nucleosides (as above) + LPV/r

Alternative to ZDV is TDF, in the case of anemia.

Alternatives to LPV/r include: ATV/r, SQR/r and FosAPV/r, if available.

Note: NVP should not be used for PEP due to risk of hepatotoxicity.

- If PEP is given, the exposed person should have a baseline Hemoglobin test, if Zidovudine is used as part of PEP.
- HIV Antibody testing (rapid or ELISA) should be used to monitor for seroconversion, and test should be performed at baseline and at 3 and 6 months post exposure.
- Testing for other bloodborne diseases- such as hepatitis B and C- is also important; depending on the nature of the risk and the local prevalence, if testing is available.

Counseling for HIV Post-exposure Prophylaxis

In the process of seeking informed consent for HIV post-exposure prophylaxis, people who have been exposed to HIV must be made fully aware of the following:

- The risk of acquiring HIV infection from the specific exposure;
- What is known and not known about the efficacy of PEP;
- The importance of taking a HIV test and of receiving appropriate post-test counseling (although testing may be delayed if necessary);
- The possibility that they might already be infected with HIV will need to be assessed if they have not already had an HIV test;
- People already living with HIV should be referred for treatment of their infection, and if they had started PEP the medicine should be stopped when the diagnosis is confirmed;
- People with discordant rapid HIV test results should be offered PEP while waiting for pending laboratory-based confirmatory testing;
- PEP medication will be discontinued if their initial HIV test is positive: this medication could increase the risk of drug resistance among people already infected;
- The importance of adhering to medicine;
- What to do if they forget or vomit a dose (see ART adherence section)
- The duration of the course of medicine (four weeks);
- The common side effects that may be experienced while taking PEP medicine;
- That they can stop taking PEP medicine at any time, but if they do so, they will probably not get the full benefit of PEP, if the source to which they were exposed was HIV positive;
- PEP medicine can be taken during pregnancy and may protect the mother from getting HIV infection after exposure;
- That continuing to breastfeed while taking PEP is safe, although if women get infected by HIV while breastfeeding, the risk of transmitting HIV through breastfeeding is higher at the early stage of infection in the absence of ARVs. All women in Nepal should exclusively breastfeed their infants for the first 6 months of life with continued breastfeeding until 12 months (if HIV infected or at least 2 years (if HIV negative).

PEP Following Sexual Assault

There are no available data about the use of PEP following rape. But if the risk of transmission of HIV is considered to be present, PEP, as used for health workers after occupational exposure, can be used taking into account various factors, including drug toxicity and adherence, as discussed above. PEP for the victim of rape can be made available if required.

PEP for Non-occupational Exposure Other Than Rape:

For non-occupational exposure other than rape, clinician will decide on a case-by-case basis whether PEP should be provided. It should not be provided in the case of chronic HIV exposure or cases of "recreational exposure". Provider may decide to provide PEP in some cases, such as an episode of condom breakage in a discordant couple.

Where should PEP be Available in Nepal?

- Drugs for PEP should be made available in every hospital so that treatment can be immediately initiated. Further management must be decided by an expert as soon as possible.
- Starter packs of between 2 and 5 days of ARVs can be placed in other medical facilities with linkages to full PEP packages. These can be prescribed under the condition that the client return to see a designated provider for complete risk assessment and to collect the rest of the 28 days of medicine. This helps prevent large wastage due to expiry of unused PEP packs.

Table 20: Clinical Management of HIV Post-exposure Prophylaxis: Summary

Item	Recommended Action and Notes
Eligibility	Exposure within 72 hours<br>Exposed individual not known to be infected with HIV<br>Significant exposure<br>Person who was the source of exposure is HIV infected or has unknown HIV status
Informed consent for post-exposure prophylaxis	Information about risks and benefits<br>Consent may be given verbally
Medicine	Two nucleoside-analogue reverse-transcriptase inhibitors (usually part of first-line antiretroviral therapy medicines)<br>Dispensed by appropriately qualified person<br>Add a boosted protease inhibitor to the regimen if higher risk exposure.
Time to initiation	The initial dose of antiretroviral medicines should be given as soon as possible but no later than 72 hours after exposure
Duration of therapy	28 days
HIV testing with informed consent and pre- and post-test counseling	Baseline HIV test in exposed person<br>Follow-up HIV testing 3 and 6 months after exposure<br>Rapid HIV test of the source person if feasible and based on informed consent
Additional laboratory evaluations	Pregnancy testing<br>Haemoglobin (for ZDV-containing PEP regimens)<br>Hepatitis B and C screening, if available and based on the prevalence of the diseases
Counseling	For adherence; side effects; risk reduction; trauma or mental health problems; and social support and safety
Referral	Referrals as appropriate
Record-keeping	Maintain accurate, confidential records
Follow-up-clinical	Assess and manage side effects<br>Assess and support adherence

XVII. Management of HIV in Infants and Children

I. Diagnosis of HIV Infection in Children

Laboratory Diagnosis of Pediatric HIV Infection

There are three types of tests available to confirm HIV infection in children and to monitor the progress of disease.

1. HIV antibody testing continues to be the backbone; however it is of more limited use in children aged <18 months, who may still be carrying passively transferred maternal HIV antibodies.

- All infants born to HIV infected mothers will test antibody positive at birth.
- However, children over 12 months of age have usually lost maternal antibody. If ELISA is positive after 12 months of age, there is a 96% chance the child is HIV-infected.
- All HIV-exposed infants should undergo virologic testing at 6 weeks of age, if available.
- All exposed infants should be tested at 9 months during the time of measles vaccination. Those tested positive should be referred to sites where DNA PCR tests are available for early infant diagnosis.

2. Virological tests are needed to confirm HIV infection in children less than 18 months of age.

- DNA PCR is the most common virological test for definitive diagnosis in infants (qualitative).
- RNA PCR (quantitative) also called Viral Load is most commonly used to follow response to ART.
- Virological tests become positive much earlier than antibody tests.
- If tested at 6 weeks almost all infants infected intrauterine and peripartum will be positive.
- Recently, Dried Blood Spot (DBS) has been found to be reliable for DNA PCR sample collection and allows finger or heel-stick collection along with ease of transportation of the sample to a central laboratory from other parts of the country.

- If DNA PCR is available, all HIV-exposed infants should be tested at 6 weeks of life. Those testing negative should have DNA PCR repeated 6 weeks after breastfeeding cessation. All infants should undergo HIV antibody testing for confirmation at 18 months of age. All positive DNA PCR tests require a second virologic test on a new sample. ART should be initiated immediately and not delayed for confirmatory results.

3. Immunological tests: (CD4 counts, CD4%) contribute enormously to care and treatment decisions.

- CD4 cell count is a good predictor of progression of HIV disease.

- CD4% is the percent of total lymphocytes that are made up of CD4 cells. This is more useful for children under 5 years of age than the absolute CD4 count because CD4 count fluctuate with concurrent illness, physiological changes and timing of test. However where CD4% facilities are not available absolute CD4 count can be determined and age specific CD4 count criteria used.

Note: Breastfeeding further complicates diagnosis in children. Antibody and virological tests must be performed at least 6 weeks after the cessation of breastfeeding for accurate diagnosis.

- Combining clinical and laboratory criteria to stage HIV disease ensures timely and rational initiation of care, treatment, and appropriate counseling.
- Definitive HIV diagnosis in children >18 months can be performed with antibody tests, following standard testing algorithms as used for adults.

Presumptive Clinical Diagnosis of HIV Infection

For infants and children aged less than 18 months where access to virologic testing is not available but a child has symptoms that are suggestive of HIV infection, a presumptive clinical diagnosis of HIV infection may need to be made as follows:

Criteria for presumptive clinical diagnosis of severe HIV disease in infants and children aged under 18 months in situations where virological testing is not available:

A presumptive diagnosis of severe HIV disease should be made if:

- Infant is confirmed HIV-antibody positive; and
- Diagnosis of AIDS-indicator condition(s)a can be made

  or
- The infant is symptomatic with two or more of the following:
  - Oral thrush
  - Severe pneumonia
  - Severe sepsis

Other factors that support the diagnosis of severe HIV disease in an HIV seropositive infant include:

- Recent HIV-related maternal death or advanced HIV disease in the mother.
- CD4 <20%.

Confirmation of the diagnosis of HIV infection should be sought as soon as possible.

II. Antiretroviral Therapy (ART) in Infants and Children

The advent of potent antiretroviral therapy has dramatically reduced rates of mortality and morbidity and has improved the quality of life of infants and children living with HIV although it does not provide a cure. As a result HIV is now perceived as a manageable chronic illness.

Indications for Starting ART in HIV-Infected Infants/Children:

Under 24 Month

- Treat all HIV-infected children under 24 months, irrespective of clinical or immunological stage.
- If child is less than 18 months of age use DNA PCR.
- Where DNA PCR is not available, ART needs should be based on presumptive clinical diagnosis of severe HIV disease for those under 18 months.

For Children Above 24 Months of Age

- Clinical status: Based on WHO Pediatric Clinical Staging
- Immunological status: CD4 Absolute Counts/CD4%

Table 21: Recommendations for Initiating ART in Children over 24 Months of Age

**Recommendations for Initiating ART in Children Above 24 Months of Age**		
WHO Paediatric Stage	Availability of CD4 Cell Measurements	Treatment Recommendation
4[a]	CD4[b]	Treat all
	No CD4	
3[a]	CD4[b]	Treat all
	No CD4	
2	CD4[b]	CD4-guided
	No CD4	Do not treat, if no CD4 available
1	CD4	CD4-guided
	No CD4	Do not treat, if no CD4 available

Notes:
a. Stabilize any opportunistic infection prior to initiation of ARV therapy
b. CD4 is useful to monitor ART, even if it is not required to initiate therapy

Table 22. Recommendations for Age-related CD4 Values for ART Decisions in Children

**Immunological Marker**	**<24 months of age**	**24-59 months**	**≥ 5yrs**
**CD4%***	All	≤25%	NA
**CD4 Count***	All	≤750 cells/mm^3	≤350 cells/mm^3

Recommended First-line Regimens for Children

Babies less than 2 years of age:

- If no or unknown PMTCT exposure to ARVs , start 2 NRTIs + NVP
- If baby or mom took NVP or EFV for PMTCT, start 2 NRTIS + LPV/r

Children 2 to 3 years of age

- Regardless of PMTCT ARV exposure, start 2 NRTIs + NVP

Children over 3 years of age

- Regardless of PMTCT ARV exposure, start either 2 NRTIs + NVP or 2 NRTIS + EFV

2 NRTIs (Nucleoside backbone) should be:

- First choice: ZDV +3TC
- 2nd choice: ABC+3TC
- 3rd choice: d4T+ 3TC

III. Immunization of Children Exposed/Infected with HIV

The majority of children with maternally transmitted HIV infection acquire the infection during or shortly after birth. Early in life they are immunologically normal and only later without specific treatment do they develop progressive immunodeficiency.

All children who have been exposed to HIV should be fully immunized according to age. Because most children who are HIV-infected do not have severe immune suppression during the first year of life, immunization should occur as early as possible after the recommended age to optimize the immune response.

BCG and live attenuated vaccines (including influenza, Japanese encephalitis, measles, mumps, rubella, typhoid, varicella and yellow fever) should not be given to children with severe immunodeficiency.

Table 23: Nepal National Immunization Schedule for HIV Exposed or Infected Infants

Age of infant	Vaccine
Birth	BCG
6 weeks	DPT1, HBV1, Hib 1, OPV1
10 weeks	DPT2, HBV2, Hib 2, OPV2
14 weeks	DPT3, HBV3, Hib 3, OPV3
6 months	Extra dose of Measles*
9 months	Measles

*Because of the increased risk of early and severe measles infection, HIV-exposed infants who are not severely immunocompromised should receive an extra dose of standard measles vaccine at 6 months of age with a second dose as soon after the age of 9 months as possible.

Optional Vaccines:

Refer to the latest edition of National guidelines for the Management of HIV in Children in Nepal.

XVIII. Antiretroviral Therapy in Injecting Drug Users

Initial Evaluation

Care for HIV-positive injecting drug users (IDU) must address substance use and substance dependence, psychological and social issues, and medical complications associated with injecting drug use and HIV.

Evaluation of Substance Use and Dependence

Standardized assessment tools should be used for screening and initially evaluating substance use and dependence. Any screening or assessment must be voluntary and fully informed, with explanation of why the service needs to understand the individual's substance use and associated problems. Under-reporting use of illicit substances is common, so all patients should be screened for substance use and dependence

Patients who admit to substance use should be examined further, as should those who do not but present with clinical signs or symptoms of drug use, including injections. It is crucial to assess drug dependency, as it has implications for patient management strategy.

Typically a substance use and dependence assessment includes a complete history of substance use and treatment and a physical examination. A substance use and treatment history will include:

- Substances used, including alcohol and combinations of drugs, and age at first use
- Modes of drug administration
- Lifetime, recent and current use
- Changes in drug effects over time
- History of tolerance, overdose and withdrawal
- Periods of abstinence and attempts to quit
- Complications of substance use (hepatitis, abscesses, etc.)
- Current problems, including severity of dependence
- Types and outcomes of previous treatment for drug dependence.

A physical examination may indicate substance dependence and/or complications associated with substance use. The physical complications of opioid or other drug dependence should be identified and addressed as part of the overall treatment

plan. Further evaluation of drug dependence severity and appropriate treatment strategy should be done by, or in close collaboration with, substance dependency treatment experts or other trained staff.

Initial Evaluation of HIV Status

Initial evaluation of IDUs' HIV status is no different from that of non-users.

Further Clinical Evaluation

Further evaluation is required for developing a strategy of clinical management of HIV-infected IDUs, including:

- Presenting symptoms
- Physical examination
- Mental health and social assessment
- Preparedness for treatment
- Routine laboratory assessments
- CD4 lymphocyte count to determine the severity of immunodeficiency
- History of contraception use and pregnancy test if indicated
- Assessment for hepatitis B and C
- Screening for TB
- Assessment for psychiatric disorders
- Weight
- Other tests based on the patient's condition.

Since many IDUs present for care at an advanced stage of HIV infection, it is important to thoroughly evaluate new patients for active opportunistic infections. The initial history and physical examination will usually identify common complications, including:

- Oral candidiasis and difficulty swallowing, suggesting oesophageal candidiasis
- Non-healing genital or anal ulcers, indicating herpes simplex
- Fever with cough and/or shortness of breath, suggesting bacterial pneumonia, TB or PCP.

Initial evaluation should be followed by treatment of opportunistic infections and other conditions as indicated.

Psychosocial Assessment

Mental health comorbidities are common among IDUs with HIV. Some estimates suggest that between 25% and 50% of drug users also have a comorbid mental health problem.

A thorough psychosocial assessment should be undertaken at initial evaluation, focusing on:

- Any source of instability that might undermine adherence to treatment
- Depression and other mood disorders
- Other psychiatric problems

Social factors to be assessed include:

- Social stability, family and community support
- Homelessness
- Major life events and crises
- Financial security
- Nutrition

Interactions between ARVs and Methadone/Buprenorphine

Methadone and buprenorphine are the most common drugs prescribed for Opioid Substitution Therapy (OST). Significant interactions with some of the most commonly used ARVs.

AZT and Methadone

AZT does not change methadone levels in the bloodstream. Methadone significantly increases the blood concentration of AZT (43%). Watch for possible increases in AZT toxicity: anaemia, myalgia, bone marrow suppression, fatigue, headache and vomiting.

EFV and Methadone

Efavirenz (EFV) can significantly decrease the concentration of methadone in the blood by 60%, and can cause methadone withdrawal. withdrawal can be delayed and possibly not seen until 2-3 weeks after starting the EFV. May require a methadone dose increase of 50%.

NVP and Methadone

Nevirapine (NVP) can significantly decrease the blood concentration of methadone (46%). Methadone withdrawal is common. withdrawal can be delayed and possibly

not seen until 2-3 weeks after starting NVP. May need a methadone dose increase of approximately 15%.

PIs and Methadone

PIs can induce or inhibit CYP3A.

- PIs can induce CYP3A causing faster metabolism of other drugs. **This will decrease** blood levels of PIs. Faster metabolism of methadone may cause withdrawal.
- PIs can inhibit CYP3A causing slower metabolism of other drugs. **This will increase** blood levels of PIs. Slower metabolism of methadone may cause toxicity.

Ritonavir and Methadone

Ritonavir (RTV) can significantly decrease methadone levels in the blood by 26-53% and can cause methadone withdrawal. withdrawal symptoms can be delayed by 2-3 weeks. Side-effects of RTV may mimic withdrawal symptoms.

ANNEX I

Antiretroviral Regimens or Components That should Not be Offered at Any Time

Antiretroviral Regimens Not Recommended	Rationale	Exception
**Monotherapy**	Rapid development of resistance Inferior antiretroviral activity when compared to combination with three or more antiretrovirals	
**Dual-NRTI Regimens**	Rapid development of resistance. Inferior antiretroviral activity when compared to combination with three or more antiretrovirals	Some cases of PEP
**Triple-NRTI Regimens Except for Abacavir/ Zidovudine/ Lamivudine or Tenofovir + Zidovudine/ Lamivudine**	High rate of early virologic non-response seen when triple NRTI combinations including ABC/TDF/ 3TC or TDF/ddI/3TC were used as initial regimen in treatment-naïve patients Other triple-NRTI regimens have not been evaluated	Abacavir/zidovudine/ lamivudine ; and tenofovir + zidovudine/ lamivudine in selected patients in whom other combinations are not desirable
**Antiretroviral Components Not Recommended as Part of Antiretroviral Regimen**		
**Tenofovir + Didanosine**	High rate of poor virologic response seen when this NRTI combination was used	
**Didanosine + Stavudine**	High incidence of toxicities- peripheral neuropathy, pancreatitis, and hyperlactatemia Reports of serious, even fatal, cases of lactic acidosis with hepatic steatosis with or without pancreatitis in pregnant women	When no other antiretroviral options are available and potential benefits outweigh the risks
**2-NNRTI Combination**	When EFV combined with NVP, higher incidence of clinical adverse events seen when compared to either EFV- or NVP-based regimen<br><br>Both EFV and NVP may induce metabolism and may lead to reductions in etravirine (ETR) exposure; thus, they should not be used in combination	No exception

Antiretroviral Regimens Not Recommended	Rationale	Exception
**Efavirenz in First Trimester of Pregnancy or in Women with Significant Child-Bearing Potential**	Teratogenic in nonhuman primates	When no other antiretroviral options are available and potential benefits outweigh the risks
**Nevirapine in Treament-naïve Women with CD4 >250 or Men with CD4 >400**	High incidence of symptomatic hepatotoxicity	If no other antiretroviral option available, if used patients should be closely monitored
**Stavudine + Zidovudine**	Antagonistic effect on HIV-1	No exception

ANNEX II

Drug Interactions with Antiretrovirals

ARVs	NVP	EFV	LPV/r
**ANTIMYCOBACTERIALS**			
Rifampicin	↓NVP level by 20% to 58%. Virological consequences are uncertain; potential for additive hepatotoxicity exists. Coadministration is recommended only if done with careful monitoring.	↓EFV level by 25%	Decreased LPV AUC by 75%. Should not be coadministered.
Rifabutin	Levels: NVP ↓ 16%. No dose adjustment.	Levels: EFV unchanged. Rifabutin ↓35%. Dose: ↑ rifabutin dose to 450-600 mg once daily or 600 mg three times a week. EFV: standard.	Levels: rifabutin AUC increase threefold. Decrease rifabutin dose to 150 mg 3 times a week LPV/r: standard dose
Clarithromycin	None	Decrease clarithromycin by 39%. Monitor for efficacy or use alternative drugs.	Decrease clarithromycin AUC by 75%. Adjust clarithromycin dose if renal impairment.
**ANTIFUNGALS**			
Ketoconazole	↑ ketoconazole level by 63%<br>↑ NVP level by 15-30%.<br>Do not recommend coadministration	No significant changes in ketoconazole or EFV levels	Increase LPV AUC. Increase ketoconazole level threefold. Do not exceed 200 mg/day ketoconazole.
Fluconazole	↑ NVP Cmax, AUC, Cmin by 100%.<br>No change in fluconazole level.<br>Possible increase in hepatotoxicity with coadministration requiring monitoring of NVP toxicity.	No data	No data

ARVs	NVP	EFV	LPV/r
Itraconazole	No data	No data	↑ itraconazole level. Do not exceed 200mg/ day itraconazole.
**ORAL CONTRACEPTIVES**			
Ethinyl estradiol	↑ ethinyl estradiol by 20%. Use alternative or additional methods.	↑ ethinyl estradiol by 37%. Use alternative or additional methods.	Decrease ethinyl estradiol level by 42%. Use alternative methods.
**ANTICONVULSANTS**			
Carbamazepine Phenytoin	Unknown use with caution. Monitor anticonvulsant levels and watch virologic response. May cause decrease in NVP levels	Use with caution. One case report showed low EFV concentrations with phenytoin	Many possible interactions. Carbamazepine: Increase levels when coadministered with RTV. Use with caution. Monitor anticonvulsant levels. Phenytoin: Decrease levels of LPV and RTV, and • Decrease levels of phenytoin when administered together. Avoid concomitant use or monitor LPV/ anticonvulsant levels.

ARVs	NVP	EFV	LPV/r
**OPIOID SUBSTITUTION TREATMENT**			
**Methadone**	Levels: NVP unchanged. Methadone Decreases significantly. Opiate withdrawal common when this combination is used. Increased methadone dose often necessary. Titrate methadone dose to effect.	Levels: methadone decrease 60%. Opiate withdrawal common, increase in methadone dose often necessary. Titrate methadone dose to effect.	Methadone AUC decrease 53%. Opiate withdrawal may occur. Monitor and titrate dose if needed. May require increase in methadone dose.
**Buprenorphine**	Not studied	Buprenorphine levels decrease 50% but no withdrawals reported. No dose adjustment is recommended.	No significant interactions
**Simvastatin, Lovastatin**	No data	Decrease simvastatin level by 58%. EFV level unchanged. Adjust simvastatin dose according to lipid response; not to exceed the maximum recommended dose.	Potential large increase statin level. Avoid concomitant use.
**Atorvastatin**	No data	↑ atorvastatin AUC by 43%. EFV level unchanged. Adjust atorvastatin dose according to lipid response; not to exceed maximum recommended dose.	Increase atorvastatin AUC 5.88 fold. Use lowest possible starting dose with careful monitoring.
**Pravastatin**	No data	No data	Increase Pravastatin AUC 33%. No dose adjustment needed.

All the PIs and EFV can increase levels of cisapride and non-sedating antihistamines (aztemizole, terfenedine), which can cause cardiac toxicity. Coadministration is not recommended.

ANNEX III

Drugs That Should Not be Used with PI or NNRTI Antiretrovirals

Drug Category	Calcium Channel Blockers	Cardiac Agents	Lipid Lowering Agents	Antimyco Bacterials	Anti-histamines	GI Drugs	Neuro-leptics	Psycho-tropics	Ergot Alka Loids (Vasocon-Strictors)	Herbs	Others
Indi-navir	(None)	Amiod arone	Simvas-tatin lovastatin	Rifampin rifapentine	astemi-zole terfe-nadine	Cisap-ride	Pimo-zide	Midazo-lam tri-azolam		St. John's wort	Ataz-anavir
Lopin-avir/ riton-avir	(None)	Flecaini-de prop-afenone	Simvas-tatin lovastatin	Rifampin rifapentine	Astemi-zole ter-fena-dine	Cisap-ride	Pimo-zide	Mida-zolam triaz-olam	As above	St.-John's wort	Fluti-casone
Efavi-renz	(None)	(None)	(None)	Rifapentine	Astemi-zole terfena-dine	Cisap-ride	(None)	Mida-zolam triaz-olam		St.-John's wort	
Nevir-apine	(None)	(None)	(None)	Rifapentine	(None)	(None)	(None)	(None)	(None)	St. John's wort	

ANNEX IV

PATIENT VISIT RECORD CARD

Name and Address of Health Institution

Patient Name or ID

Hospital/Clinic Number | Date of visit

Patient History:

- HIV related disease including TB
- Cough
- Fever
- Weight Loss
- Diarrhea
- Other symptoms of GI, CNS, Skin

Other Medications: (If Any)

Drug Allergies:

WHO Staging:

Is There Any Change Since Last Visit?

Adherence to antiretroviral therapy:

No of doses missed in last 7 days:

No doses missed since last visit:

Dose taken at correct time:	yes	No
Correct taken:	yes	No
Dose delay >1 hr:	yes	No

Specify reason for interruption or modification/failure to take prescribed doses:

Other Medications: New and ongoing (if new, indicate Start date)

Medication	Start Date	COMMENTS

Body Weight:.................. kg Pulse.......... BP.................... Temp............ ° C

Resp. Rate.......................

PHYSICAL EXAMINATION: (Tick if Normal, Describe If Abnormal)

General condition ....

Skin ....

ENT ....

Lymph nodes ....

Heart ....

Chest ....

Abdomen ....

GU Tract ....

Musculoskeletal system ....

Extremity ....

Neuro logical system ....

Other (describe) ....

HIV-RELATED ILLNESS: New and ongoing (if new, indicate Start Date)

Are there any new HIV-related illness at this visit? No .... Yes (if yes, specify) ....

**COMMENTS**	**START DATE**
■ Oral candidiasis	/ /
■ Oral hairy leukoplakia	/ /
■ Pruritic popular eruption	/ /
■ Lymphadenopathy (>1 cm on both sides)	/ /
■ Other HIV related illnesses	/ /

BASIC LABORATORY RESULTS

Hemoglobin	g/dl WBC	cells/cumm	
Platelets	cells/cumm		
Total lymphocyte count		cells/cumm	
Glucose	mg/dl	Creatinine	mg/dl
ALT/SGPT	U/l	RFT	

Other lab results (e.g. CXR, AFB, culture, serology)

NOTES/PLAN

....

....

....

....

....

....

....

....

....

....

....

....

....

Follow-up Date:

(A booklet with about 12 copies of the above made for each patient for monitoring is recommended).

ANNEX V

PEP Form

Occupational Exposure Report

**Name:**	Address (workplace):<br><br>Address (home):

Birth date:	Sex:	Position:	Telephone No.

Date/time of exposure:	Location exposure occurred:

Activity at time of exposure:

Nature of injury (e.g., needle stick, cut, splash):

Details of the procedure being performed, including where and how the exposure occurred:

Details of the exposure, including the type and amount of fluid or material and the severity of the exposure

Details about the exposure source:

The source material contained: HBV:

HCV:

HIV:

Is the source HIV-infected:

Stage of disease:

History of antiretroviral therapy:

Antiretroviral resistance:

Pre-test counseling provided:

Results of the tests:

HIV

Post-test counseling provided:

Referral:

Details about The Exposed Person:

Infected with: HBV:

HCV:

HIV:

Concomitant diseases:

Hepatitis B vaccination:

Vaccine-response status:

Pre-test counseling provided:

Results of the tests:

HIV

Post-test counseling provided:

Referral:

PEP prophylaxis commenced:

Drugs provided:

Counseling on protecting others: avoiding unprotected intercourse and breastfeeding

Postexposure management:	Hb
Baseline visit	
HIV Antibody test results:<br>3 month<br>6 month	

**Signature:**	Date:

ANNEX VI

Antiretroviral Drugs Pediatric Dose Chart- Nepal 2011

	Zidovudine/Lamivudine/Nevirapine (ZDV+3TC+NVP)		Zidovudine/Lamivudine/ (ZDV+3TC)		Stavudine/Lamivudine/ Nevirapine (d4T+3TC+NVP)	Stavudine/Lamivudine/ (d4T+3TC)	Zidovudine (ZDV/AZT)	Lamivudine (3TC)
	ZDV 180-240mg/m²/dose<br>3TC 4mg/kg/dose<br>NVP 160-200mg/m²/dose<br>**All Twice Daily**<br>(Note: NVP only once daily for 1st 14 days)<br>Prefer Pediatric Tablet		ZDV 180-240mg/m²/dose<br>3TC 4mg/kg/dose<br>**Both Twice Daily**<br>(Note: Give with either NVP, EFV or LPV/r)		d4T 1mg/kg/dose<br>3TC 4mg/kg/dose<br>NVP 160-200mg/m²/dose<br>**All Twice Daily**<br>(Note: NVP only once daily for 1st 14 days)	d4T 1mg/kg/dose<br>3TC 4mg/kg/dose<br>**Both Twice Daily**<br>(Note: Give with either NVP, EFV or LPV/r)	240mg/m²/dose<br>**Twice Daily**	4mg/kg/dose<br>**Twice Daily**
Weight	Pediatric Tablet (Dispersable) ZDV+3TC+NVP (60mg/30mg/50mg)	Adult Tablet ZDV+3TC+NVP (300mg/150mg/200mg)	Pediatric Tablet (Crushable) ZDV+3TC 60mg/30mg	Adult Tablet ZDV+3TC 300mg/150mg	Pediatric Tablet (Dispersable & Crushable) d4T/3TC /NVP (6mg/30mg/50mg) Twice Daily	Pediatric Tablet (Dispersable & Crushable) d4T/3TC 6mg/30mg	Capsule ZDV 300mg	Capsule 3TC 150mg
3-5 kg	1 BD	-	1 BD	-	1 BD	1 BD	-	-
6-9 kg	1½ BD	-	1½ BD	-	1½ BD	1½ BD	-	-
10-13 kg	2 BD	-	2 BD	-	2 BD	2 BD	-	-
14-19 kg	2½ BD	½ BD + ½ extra NVP (200mg) PM	2½ BD	½ BD	2½ BD	2½ BD	½ BD	½ BD
20-24 kg	3 BD	1 AM ½ PM	3 BD	1 AM ½ PM	3 BD	3 BD	1 AM ½ PM	1 AM ½ PM
25-35 kg	-	1 BD	-	1 BD	-	5 BD	1 BD	1 BD

ANNEX VI

Antiretroviral Drugs Pediatric Dose Chart- Nepal 2011

Weight	Nevirapine (NVP)	Efavirenz (EFV)		Abacavir (ABC)		Didanosine (DDI)		Lopinavir/ritonavir (LPV/r)		Tenofovir (TDF)
	Induction dose **Once Daily** for 14 days<br>Maintenance dose 160-200mg/m^2 dose **Twice Daily**	Dose: 15mg/kg >3yrs and 10kg **Once Daily**		8mg/kg dose **Twice Daily**		90-120mg/m^2/dose **Twice Daily >5kg**		Doses of LPV:<br>5-7 kgs: 16mg/kg/dose<br>8-9 kgs: 14mg/kg/dose<br>10-13 kgs: 12mg/kg/dose<br>14-39 kgs: 10mg/kg/dose<br>**Twice Daily**		Only for Children> 12 yrs<br>300mg **Once Daily**
	Tablet NVP 200mg	Capsule EFV 200mg	Capsule EFV 600mg	Pediatric Tablet (Crushable) ABC 60mg	Adult Tablet ABC 300mg	Pediatric Tablet (Dispersable, Crushable, chewable) DDI 25mg	Adult Tablet DDI 250mg	Pediatric Tablet (Do not crush or chew) LPV/r 100mg/25mg	Tablet LPV/r 200/50mg	Tablet TDF (Do not crush, cut or chew) 300mg
3-5 kg	-	-	-	1 BD	-	2 BD (between 5 and 6 kg)	-	-	-	Do not use in children <12 years old
6-9 kg	-	-	-	1 ½ BD	-	3 AM 2 PM	-	2 AM 1 PM	-	
10-13 kg	-	1 OD	-	2 BD	-	3 BD	-	2 AM 1 PM	-	
14-19 kg	1 AM ½ PM	1 ½ OD	½ OD	2 ½ BD	½ BD	4 AM 3 PM	-	2 BD	1 BD	
20-24 kg	1 AM ½ PM	1 ½ OD	½ OD	3 BD	1 AM ½ PM	4 BD	1 OD	2 BD	1 BD	1 OD
25-35 kg	1 BD	2 OD	-	-	1 BD	5 BD	1 OD	3 BD	2 AM 1 PM	1 OD

ANNEX VI

Antiretroviral Drugs Pediatric Dose Chart- Nepal 2011

Zidovudine- Tablets can be crushed and capsules may be opened and dispersed in water or onto a small amount of food and immediately ingested. Oral solution is stable at room temperature but light sensitive. Do not use with Stavudine due to antagonistic effect. Use with caution in children with anemia.

Lamivudine- Tablets can be divided into two equal halves and crushed and dispersed in water or onto a small amount of food and immediately ingested. Oral solution is stable at room temperature. Use within one month of opening.

Lamivudine- Capsules may be opened and dispersed in water or onto a small amount of food and immediately ingested. The oral solution needs to be refrigerated and shaken well before use. Do not use with Zidovudine due to antagonistic effect.

Abacavir- It is available as a tablet or a yellow oral solution. The solution and tablets can be stored at room temperature.

Tablets can be divided onto two equal halves and crushed and dispersed in water or onto a small amount of food and immediately ingested It can cause severe hypersensitivity reactions and should never be used if it occurs.

Didanosine- It should be given on an empty stomach one hour before or two hours after a meal. The suspension needs to be refrigerated and shaken well before administering.

At least two tablets of appropriate strength must be used at any one time for adequate buffering (e.g. If the dose is 50mg, administrator two 25 mg tablets should be chewed, crushed or dispersed in water before they are taken. Capsules are designed for once daily dosing.

Nevirapine- Tablets can be divided into two equal halves, crushed and dispersed in water or onto small amount of food and immediately ingested. Oral solution is stable at room temperature.

Shake well before use. Rash can occur during the first 14 days of closing. If severe rash occurs (especially if accompanied by fever, blisters or mucosal ulcerations), discontinue drug. Avoid coadministration with Rifampicin if possible.

Efavirenz- It is not approved for children <3 years and below 10kg weight. It can be given with or without food, but high fat meals should be avoided for best absorption. Preferably given at bed time to reduce CNS side effects, especially during first two weeks. Capsules can be opened and added to small amount of sweet food or drink to disguise peppery taste.

Lopinavir/ritonavir- Oral solution should be taken with food. Oral solution should be refrigerated. Oral solution is bitter to taste. No food restriction with tablets. Tablets cannot be split.

Annex VII

Early Warning Indicators

- On-Time ARV Pick-Up (EWI 4a)- Measures the proportion of patients who have picked up all their prescribed ARV drugs on time for two consecutive drug pick-ups after a baseline pick-up.

- Retention on ART at 12 months- Measures the proportion of adults and children known to be alive and on treatment 12 months after initiation of ART

This data is collected for all patients on ART (i.e. a census of all patients on ART at a site/region/country level) hence no sample size calculation is necessary. This measurement of retention can be incorporated into EWIs.

Suggested Target:

Poor Performance <75%

Fair Performance 75-84%

Desirable Performance ≥85%

- ARV drug supply continuity- number of months (or bi-monthly periods) in the designated year in which there were no stock-out days of any ARV drug routinely used at the site.

Modification to the definition above by including "bi-monthly periods" to be consistent with current reporting in Nepal about ARV stock to NCASC

Suggested Target: 100%

- ART prescribing practices- Measures the proportion of patients initiating ART at the site who are prescribed, or who initially pick up from the pharmacy, an appropriate first-line ART regimen.

The definition of an appropriate first-line regimen is dictated by the NCASC in the ART guideline, about what constitutes appropriate first-line therapy. In addition to assessing prescribing practices per the country recommendations of appropriate first-line, the data can also be analysed in the context of which regimens are most predictive of the development of HIVDR (i.e. whether any mono- or dual-therapy is being prescribed or picked-up).

Suggested Target: 100%

ANNEX VIII

National ART Programme Monitoring

1. Monitoring and Evaluation of the ART Programme with the increasing access to antiretroviral treatment (ART), a strong monitoring system is required at facility, district, provincial, national and international levels.

At Facility Level, The Objectives of Programme Monitoring are to:

- Support patient management by regularly recording and storing of key individual information for lifelong care and follow-up;
- Facilitate an accurate patient tracking system to identify those missing or lost to follow-up; and
- Support drug supply management at the facility.

At All Levels, Programme Monitoring will Help to:

- Document the progress in equitable access to HIV care and ART programmes; and
- Identify the successes and gaps over time and modify the programmes accordingly.

2. ART Program Performance Indicators at national/ international levels

2.1 ART program performance indicators at national level

The following indicators, based on the monitoring and evaluation framework were developed for national programmes to demonstrate progress in scaling up ART programmes:

Monitoring and evaluation framework

Input Indicators

1. Existence of national policies, strategy and guidelines for ART programmes.

Process Indicators

2. Percentage of districts or local health administration units with at least one health facility providing ART services in line with national standards.
3. Percentage of ARV storage and delivery points experiencing stock-outs in the preceding 6 months.
4. Number of health workers trained on ART delivery in accordance with national or international standards.
   Output indicators
5. Percentage of health facilities with systems and items to provide ART services.
6. Percentage of health facilities with ART services that also provide comprehensive care, including prevention services, for HIV-positive clients.

Outcome Indicators

7. Percentage of adults and children with advanced HIV infection receiving antiretroviral therapy
8. Percentage of patients initiating antiretroviral therapy at the site during a selected period who are taking an appropriate first line regimen 12 months later.

Impact Indicators

9. Percentage of adults and children with HIV known to be on treatment 6, 12, 24, 36, 48 months after initiation of antiretroviral therapy

2.2 Indicators at the facility level the following indicators are recommended to be produced at the facility level.

3. Standardized recording and reporting system to generate the above listed indicators, it is important to have a uniform data collection and reporting system. Standard recording and reporting ensures that key information gets stored. This helps in:

   - Easily retrieval by care providers to get an overview of the patient's progress over time;
   - Exchange of information between the different health care providers (such as, doctor, nurse, counselor, psychologist) as well as with other ART centres when the patient is referred or transferred to another clinic; and
   - Facilitate compilation and comparison of indicators at province, national and international levels.

4. List of records and reports at the facility

Recording Forms

1. Patient HIV Care/ART Record
2. Pre-ART Register
3. ART Register
4. ARV Drug Dispensing Register
5. ARV Drug Stock Register

Reporting Forms

1. ART Monthly Report
2. Cohort Analysis Report

Following Figure is The Recording and Reporting Forms in a Paper Based Monitoring System

FORM 1- PATIENT HIV CARE and ANTIRETROVIRAL TREATMENT (ART) RECORD

(To be stored in a locked cabinet at the health centre and arranged serially by registration number)

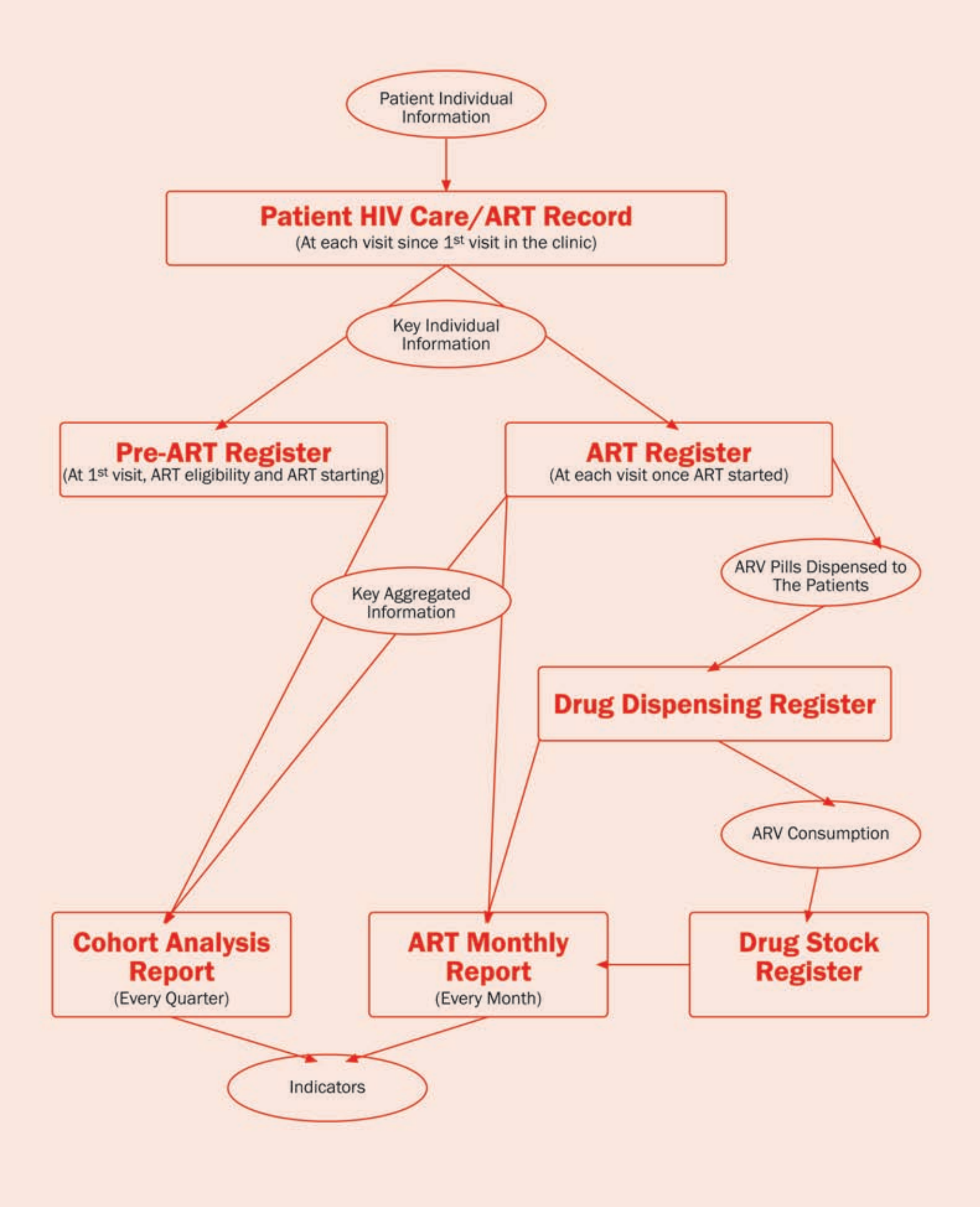

Quick Guide for Recording and Reporting Formats

Form	What Information?	For what Purpose?	When to Complete?	Who will Complete
**Patient HIV Care/ ART Record**	Demographic, HIV care, Antiretroviral treatment and monthly follow-up clinical information	■ Patient management: to ensure appropriate lifelong follow-up<br>■ Patient monitoring: to obtain key individual variables for future analysis	■ At each patient visit, starting from the 1st visit to the clinic	■ Health care providers during each patient visit
**Pre-ART Register**	Standardized and systematic key variables on each patient before ART started	■ Patient monitoring: to report key variables on each patient<br>■ Programme monitoring: to facilitate calculation of indicators	■ At the 1st visit<br>■ At start of tuberculosis treatment and cotrimoxazole prophylaxis<br>■ At ART eligibility<br>■ At start of ART<br>■ At end of follow up, if needed	■ Health care providers during each patient visit<br>Or<br>■ Trained staff using patient record after the Visit
**ART Register**	Standardized and systematic key variables on each patient under ART	■ Patient monitoring: to report key variables on each patient<br>■ Programme monitoring: to facilitate calculation of indicators	■ At each visit once ART is started	■ Health care providers during each patient visit<br>Or<br>■ Trained staff using patient record after the Visit

Form	What Information?	For What Purpose?	When to Complete?	Who will Complete
**ARV Drug Dispensing and Stock Registers**	Drugs ansd no. of tablets dispensed<br>Drug stocks	■ Patient monitoring: accounting for no. of tablets dispensed<br>■ Programme monitoring: drug consumption and available stocks	■ At the time of drug dispensing to each patient<br>■ Daily basis	Pharmacist or officer in charge of dispensing drugs
**Monthly ART Report**	Indicators	■ Programme monitoring: to calculate and analyse indicators	■ Every month	■ Facility manager<br>Or<br>■ Trained staff under supervision of the facility Manager
**Cohort Analysis Report**	Indicators	■ Programme monitoring: to calculate and analyse indicators at 6,12, 24 months of start of ART	■ Every 6 months or during yearly assessment	■ Facility manager<br>Or<br>■ Trained staff under supervision of the facility manager

Following are the reporting and recording forms to be used for ART routine program monitoring.

REFERENCES

1. WHO Guidelines for the use of Antiretroviral Treatment in Adults and Adolescents with HIV/AIDS in the South-East Asia Region, July 2002.

2. UNAIDS, WHO, AIDS epidemic update: December 2001. Geneva, Joint United Nations Programme on HIV/AIDS, 2001.

3. British HIV Association Writing Committee 2002. British HIV Association (BHIV-A) guidelines for the treatment of HIV infected adults with antiretroviral therapy. (www.aidsmap.com/bhiva.bhivagd1299)

4. Cipla Pharmaceuticals, Mumbai, India. Personal communication, 2002.

5. World Health Organization. Scaling up antiretroviral therapy in resource limited settings: Guidelines for a public health approach. Executive summary. WHO/HIV/2002.01.

6. World Health Organization. Scaling up antiretroviral therapy in resource-limited settings: Guidelines for a public health approach. WHO/HIV/2002.02.

7. British National Formulary. British Medical Association. London. UK. March 2002.

8. World Health Organization. Initiative in HIV/AIDS and Sexually Transmitted Infections. Safe and effective use of antiretroviral treatments in adults with particular reference to resource limited settings. WHO/HIS/2000; 4:23-3.

9. Ministry of Public Health, Thailand. National guidelines for the clinical management of HIV infection in children and adults. 6th ed. 2000.

10. World Health Organization and UNAIDS. Guidance modules on antiretroviral treatment. Module 5. Laboratory requirement for the safe and effective use of antiretrovirals. WHO/ASD/98.1. UNAIDS/98.7.

11. Manual of 4th Inter country workshop on clinical management of HIV/AIDS SEARO, Bangkok, WHO- July/Aug. 2002.

12. Guidelines for the use of antiretroviral agent in HIV infected adults and adolescents, DHHS, USA December 1, 2009.

13. Guidelines for Clinical Management of HIV/AIDS, India.

14. Katz MH, Hollander H. HIV infection In: Tierney LM, Mc Phee Sj. Papadakis MA, eds. Current Medical Diagnosis and Treatment 2002. New York. Large Medical Books/Mcgraw-Hill 2002:1323-53.

15. Fauci As, Lance HC. HIV Disease: AIDS and related disorders, In: Braunwald E. Fauci AS, Kasper D2, Mauser S2, Langu DL, Jameson J2., eds. Harrison's Principles of Internal medicine. New York: Mc Graw-Hill 2001: 1852-1913.

16. Tantisiriwat W, Tebas P. HIV infection and AIDS. In: Ahya SN, Flood K. Paranjothi S, eds. The Washington Manual of Medical Therapeutics. Philadelphia: Lippincott William and Wilkins 2001: 327-40.

17. Suvedi B.K. et al, Nepal Medical Association, NMA Guide Book on HIV AIDS case and Prevention, NMA: 2000.

18. Bhattarai M D. Wait AIDS is your own problem as well. Kathmandu: General Welfare Pratisthan. 1997.

19. The Johns Hopkins Hospital 2002 guide to medical care of patients with HIV Infection 10th edition.

20. WHO Scaling up Anti Retroviral Therapy in resource- limited settings: Treatment Guidelines for a Public Health Approach. Geneva 2004

21. 2005 Revision of WHO ARV Treatment Guidelines for Adult and Adolescents in Resource-Limited Settings: Meeting report. Montreux, Switzerland. June 2005.

22. World Health Organization. HIV AIDS treatment and care: treatment protocols for the WHO European region. 2007.

23. Who ART guidelines for adults and adolescents- topics for discussion. 2009 revision. May 2009

24. Prioritizing Second Line Antiretroviral Drugs for Adults and Adolescents. WHO 2007

25. Antiretroviral therapy for HIV infection in adults and adolescents in resource-limited settings: towards universal access. Recommendations for a public health approach. World Health Organization. 2006

26. WHO Rapid advice Antiretroviral therapy for HIV infection in adults and adolescents. Nov 2009

27. WHO. Rapid advice Use of antiretroviral drugs for treating pregnant women and preventing HIV Infection in infants. Nov 2009

28. WHO Antiretroviral Therapy for HIV Infection in Adults and Adolescents: Towards Universal Access: Recommendations for a public health approach. 2010

29. WHO Antiretroviral Therapy for HIV Infection in Infants and Children: Towards Universal Access: Recommendations for a public health approach. 2010

30. WHO. Antiretroviral Drugs for Treating Pregnant Women and Preventing HIV Infections in Infants: Recommendations for a public health approach. 2010

31. WHO Guidelines on HIV and Infant Feeding: Principles and recommendations for infant feeding in the context of HIV and a summary of evidence. 2010

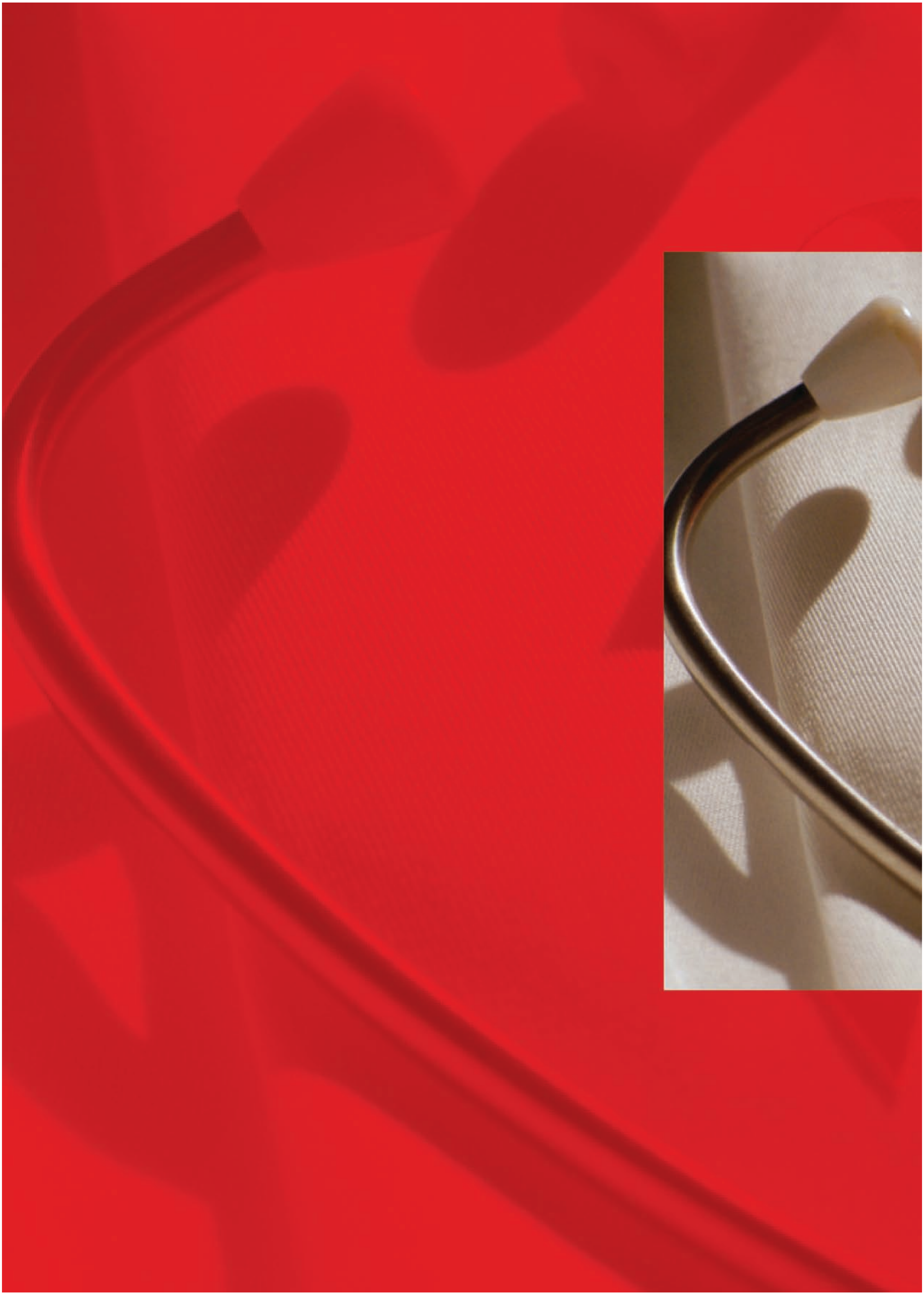

National Antiretroviral Therapy Guidelines

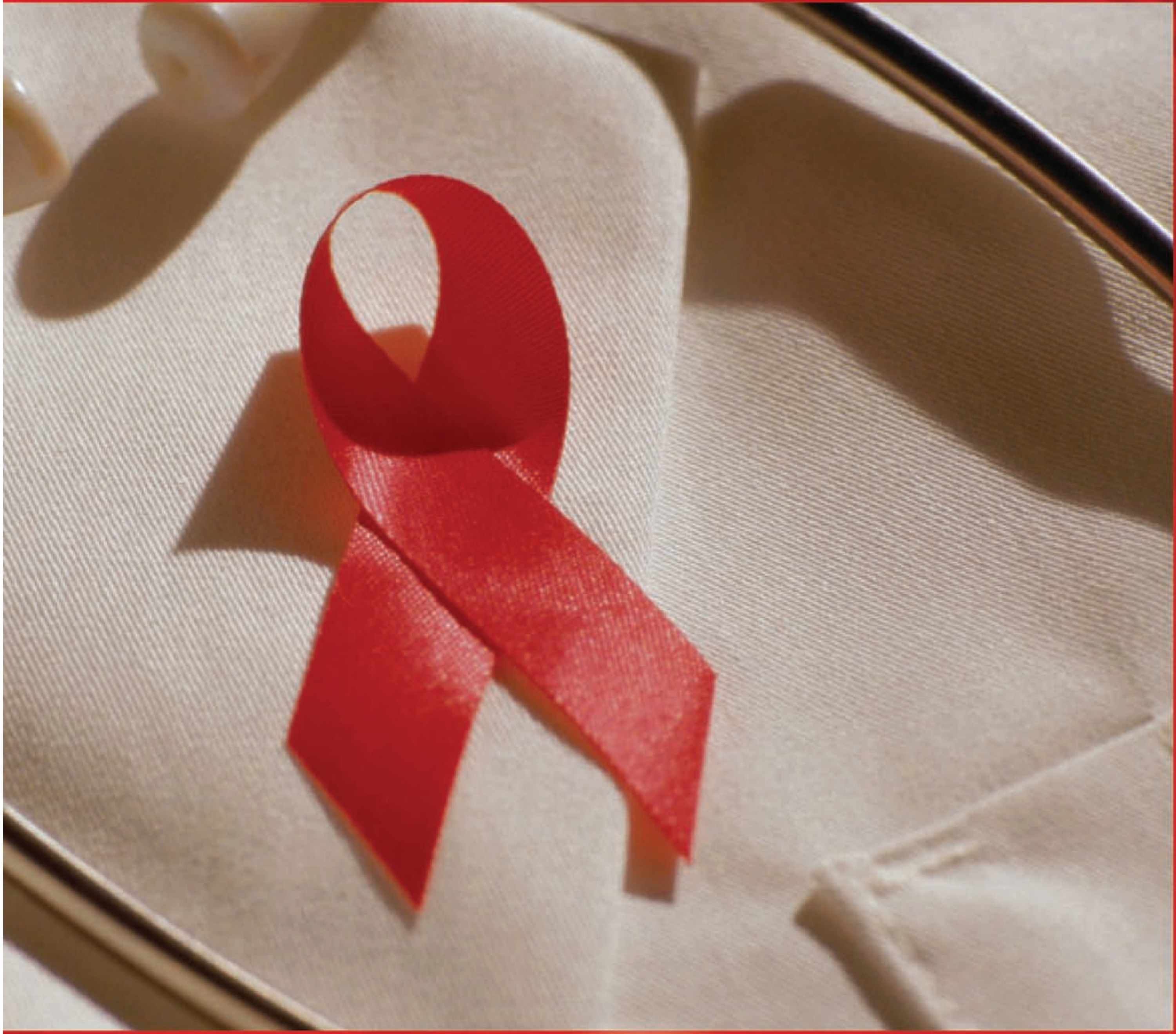

Government of Nepal
Ministry of Health and Population
National Centre for AIDS and STD Control (NCASC)
Teku, Kathmandu, Nepal
May, 2012
